# Human Milk Oligosaccharides and Lactose Differentially Affect Infant Gut Microbiota and Intestinal Barrier In Vitro

**DOI:** 10.3390/nu14122546

**Published:** 2022-06-19

**Authors:** Jane Mea Natividad, Benoît Marsaux, Clara Lucia Garcia Rodenas, Andreas Rytz, Gies Vandevijver, Massimo Marzorati, Pieter Van den Abbeele, Marta Calatayud, Florence Rochat

**Affiliations:** 1 Nestlé Institute of Health Sciences, Société des Produits Nestlé S.A., Vers-Chez-Les-Blanc, CH-1000, Lausanne 26, 1000 Lausanne, Switzerland; janemeam.natividad@nestle.com (J.M.N.); clara_garcia@bluewin.ch (C.L.G.R.); andreas.rytz@rdls.nestle.com (A.R.); florence.rochat@rdls.nestle.com (F.R.); 2 ProDigest BV, Technologiepark-Zwijnaarde 82, 9052 Ghent, Belgium; gies.vandevijver@prodigest.eu (G.V.); massimo.marzorati@prodigest.eu (M.M.); pieter.vandenabbeele@telenet.be (P.V.d.A.); marta.calatayudarroyo@gmail.com (M.C.); 3 Center for Microbial Ecology and Technology (CMET), Faculty of Bioscience Engineering, Ghent University, Coupure Links 653, 9000 Ghent, Belgium

**Keywords:** human milk oligosaccharides, infant gut microbiota, lactose, mucosal simulator of the human intestinal microbial ecosystem, prebiotic, gut microbiota, intestinal barrier integrity, permeability, inflammation, in vitro

## Abstract

Background: The infant gut microbiota establishes during a critical window of opportunity when metabolic and immune functions are highly susceptible to environmental changes, such as diet. Human milk oligosaccharides (HMOs) for instance are suggested to be beneficial for infant health and gut microbiota. Infant formulas supplemented with the HMOs 2′-fucosyllactose (2′-FL) and lacto-N-neotetraose (LNnT) reduce infant morbidity and medication use and promote beneficial bacteria in the infant gut ecosystem. To further improve infant formula and achieve closer proximity to human milk composition, more complex HMO mixtures could be added. However, we currently lack knowledge about their effects on infants’ gut ecosystems. Method: We assessed the effect of lactose, 2′-FL, 2′-FL + LNnT, and a mixture of six HMOs (HMO6: consisting of 2′-FL, LNnT, difucosyllactose, lacto-N-tetraose, 3′- and 6′-sialyllactose) on infant gut microbiota and intestinal barrier integrity using a combination of in vitro models to mimic the microbial ecosystem (baby M-SHIME^®^) and the intestinal epithelium (Caco-2/HT29-MTX co-culture). Results: All the tested products had bifidogenic potential and increased SCFA levels; however, only the HMOs’ fermented media protected against inflammatory intestinal barrier disruption. 2′-FL/LNnT and HMO6 promoted the highest diversification of OTUs within the *Bifidobactericeae* family, whereas beneficial butyrate-producers were specifically enriched by HMO6. Conclusion: These results suggest that increased complexity in HMO mixture composition may benefit the infant gut ecosystem, promoting different bifidobacterial communities and protecting the gut barrier against pro-inflammatory imbalances.

## 1. Introduction

The early postnatal period is considered a critical window of opportunity in which the assembly of microbial communities within the gastrointestinal tract significantly influences the immune, endocrine, and metabolic homeostasis [1]. Early-life colonizers of the neonatal gut are facultative anaerobes such as *Escherichia coli* and *Streptococcus* spp. that generate an optimal niche for successive colonization by obligate anaerobes such as *Bacteroides* spp., *Bifidobacterium* spp., and *Clostridium* spp. [2]. Bifidobacteria are established within the first few days after delivery and constitute up to almost 80% of the gut microbiota composition during infancy [3,4], highlighting their role as a key player in establishing a stable and resilient ecological niche. Higher copy numbers of bifidobacteria have been reported in the fecal samples of exclusively breastfed infants compared to formula-fed infants [5]. Deficient, delayed, or altered colonization of bifidobacteria populations is a common feature described in inflammatory, autoimmune, and metabolic diseases [6,7]. Thus, infant diet plays a key role in modulating microbial establishment and further improvement of infant formula composition is needed to better stimulate bifidobacterial growth.

Human milk oligosaccharides (HMOs) are specific breast milk components with multiple beneficial properties for infant health and are believed to participate in the favorable microbiota modulation by human milk. To date, there are more than 200 different HMOs described, highlighting their rich structural diversity, yet 20–25 account for >95% of total HMOs [8]. In human milk, HMOs are present in significant levels, with the highest amount described in human colostrum (up to 25 g/L) [9] and lower (but variable) levels in mature human milk, which depend on multiple factors such as maternal genetics, lactation period, and premature delivery [10]. The most abundant HMO in the breastmilk of secretor mothers is 2′-FL, an HMO that is absent in non-secretor milk. It constitutes around 30% of total HMOs and positively correlates with the concentration of LNnT [11].

HMOs are undigestible in the upper gastrointestinal tract and reach the colonic environment intact to supply substrates for the gut microbes [12,13,14], consequently impacting infant development and immune programming [9,12]. The interaction between HMOs, the host, and microbiota can be partially explained by the effect of microbial metabolites derived from HMOs that significantly affect intestinal homeostasis, such as short-chain fatty acids (SCFA) and lactate production. Bacterial metabolites are also critical mediators for gut integrity and the energy supply of epithelial cells [15]. In addition, HMOs can also act as decoy receptors, preventing pathogen attachment to infant mucosal surfaces, and can directly modulate epithelial and immune responses [9,16,17].

Breastfeeding is the best nutritional recommendation for infants during the first four to six months of age. Despite this, the World Health Organization (WHO) has reported that Europe has low (<25%) exclusive breastfeeding rates at six months of age [18,19]. Functional formulas including HMOs can be a strategy to supply non-breastfed infants with essential nutrients for developing and promoting beneficial microbial successions [20]. The European Union (EU) and the American Food and Drug Administration (FDA) have accepted the use of two HMOs, 2′-fucosyllactose (2′-FL) and/or lacto-N-neotetraose (LNnT) in infant formula [21,22,23,24]. Intervention studies demonstrate that the addition of 2′-FL and LNnT to infant formula is well-tolerated, stimulating age-appropriate growth, and triggering bifidobacterial growth and bacterial metabolic shifts similar to that of breastfed infants [25]. Specifically, a higher abundance of *Bifidobacterium* and lower representation of *Escherichia* and *Peptostreptococcaceae* were observed. HMO supplementation in infant formula also translated into significantly fewer parental reports of bronchitis and reduced use of antipyretics and antibiotics, with protective effects extended beyond the six months of intervention [26]. Overall, these data highlight that HMO supplementation could be a strategic target to improve the health of formula-fed infants.

The potential benefits of HMOs are still largely underexplored, such as their effects on mucosa-associated microbiota. In addition, HMOs in breast milk are not limited to 2′-FL and LNnT [9]. Diversifying further HMO composition in infant formula may lead to greater health effects [27] by stimulating a broader range of healthy bacterial species. We thus decided to compare the effects of lactose (the most abundant carbohydrate in human milk), 2′-FL, the well-known combination of 2′-FL/LNnT, and a more complex mix of six HMOs (HMO6) containing 2′-FL, LNnT, difucosyllactose, lacto-N-tetraose, 3′- and 6′-sialyllactose on the infant gut microbiota in vitro. Indeed, we recently showed that non-fermented HMO6 protected the epithelial barrier against inflammatory challenges (TNF-α, IFN-γ) in vitro. Little is known, however, about the effects of fermentation by the infant microbiome on these beneficial properties [28]. Therefore, we used the recently developed baby M-SHIME^®^ system [29] as it allows a long-term study (i.e., 5 weeks in the present work) of both the lumen- and the mucosa-associated bacteria together with longitudinal (proximal and distal colon) ecological differences [30]. Furthermore, the effect of bacterial metabolites derived from HMO fermentation by infant gut bacteria on intestinal barrier integrity was assessed using the baby M-SHIME^®^ culture supernatant and an in vitro model that mimics the intestinal epithelium (Caco-2/HT29-MTX co-culture).

## 2. Materials and Methods

### 2.1. Test Products

All chemicals were obtained from Merck (Darmstadt, Germany) unless stated otherwise. The 2′-fucosyllactose (2′-FL), lacto-N-neotetraose (LNnT), lacto-N-tetraose (LNT), difucosyl-lactose (diFL), 3′- and 6′-sialyllactose (3′SL, 6′SL) used in this study were provided by Glycom A/S {Horsholm, Denmark; purity > 94% (*w*/*w*)}, while lactose was acquired from Oxoid (Aalst, Belgium).

#### Sample Collection and Donor Description

Infant donors were selected based on the following inclusion criteria: healthy, age between 2–4 months, exclusively breastfed, no antibiotics or any other drug intake, no constipation or hospital-borne, and no pre-or probiotic intake. Samples were obtained from infant diapers and immediately transferred to a recipient containing an “Oxoid™ AnaeroGen™” bag to limit the samples’ exposure to oxygen. Samples were immediately transferred to the lab for further use in the short- and long-term studies. 

### 2.2. Experimental Design of Short-Term Incubations 

A short-term batch assay was performed to assess the effect of a single dose of lactose, 2′-FL, 2′-FL/LNnT, and a mixture of six human milk oligosaccharides (2′-FL, LNT, LNnT, diFL, 3′FL, 6′FL) (HMO6) on the gut microbiota composition and activity of five healthy infants. The objectives of this assay were: (i) to evaluate the inter-individual variability, and (ii) to identify a responder donor to the four treatments to further perform a long-term baby M-SHIME^®^. The criteria for selecting one donor for baby M-SHIME^®^ was based on an intermediate profile between lactose and control condition and a similar metabolic profile between different treatments in the short-term experiments. With this approach, we wanted to test if repeated administration in a long-term baby M-SHIME^®^ setup could further affect metabolic profiling and microbiota composition of an infant sample without extreme differences between lactose and HMOs in the short-term experiment. 

The incubation approach was identical to the one recently described [31], with the following modifications: 63 mL of colonic background medium (K_2_HPO_4_ 3.6 g/L; KH_2_PO_4_ 10.9 g/L; NaHCO_3_; 2 g/L; yeast extract 2 g/L; peptone 2 g/L; mucin 1 g/L; L-cysteine 0.5 g/L; polyoxyethylene (20) sorbitan monooleate 2 mL/L) was mixed with 5 g/L of lactose, 2′-FL, 2′-FL/LNnT or HMO6. Selection of dose was based on previous research reporting an estimated daily consumption of HMOs varying between 2–22 g/day at 5 months, 0–16 g/day at 9 months, and 2–50 g/day at 12 months [32]. The dose was adjusted to the low range to avoid extreme responses not representative of standard HMOs daily intake and lactose was kept at the same levels for comparison purposes.

A control condition containing a colonic medium was run in parallel and all the conditions were tested in triplicate. Then, the reactors were sealed and anaerobiosis was obtained by continuous flushing of the headspace with N_2_ for 10 min. Subsequently, freshly collected fecal samples from five healthy infants (3 months old; exclusively breastfed; D1–5) were homogenized in an anaerobic phosphate buffer (K_2_HPO_4_ 8.8 g/L; KH_2_PO_4_ 6.8 g/L; sodium thioglycolate 0.1 g/L; sodium dithionite 0.015 g/L, L-cysteine 0.5 g/L) in a proportion of 7.5% (*w*/*v*) and 1 mL of the fecal suspension was inoculated in the reactors containing colonic medium with different tested products.

Samples were collected at 0 h, 24 h, and 48 h for microbial metabolic activity analysis including pH, gas production, SCFA, lactate, and branched-chain fatty acid (BCFA) production. Additionally, at 48 h, *Bifidobacterium* levels were quantified via qPCR. 

### 2.3. Long-Term Baby M-SHIME^®^

From the short-term assay, one donor was selected to perform a long-term experiment using the mucosal simulator of the human microbial ecosystem baby M-SHIME^®^ (ProDigest and Ghent University, Ghent, Belgium), specifically adapted to mimic the infant gut (baby M-SHIME^®^), as previously described by Van den Abbeele et al., 2021 [29], with small modifications. The baby M-SHIME^®^ feed was adapted to simulate the colonic composition of a baby between 2–4 months old and contained yeast extract 1 g/L, mucin 4 g/L, L-cysteine 0.2 g/L, and digested infant formula composed of lactose 4.8 g/L, casein 0.5 g/L, and lactalbumin 4.6 g/L. The feed was mixed with 10 g/L of lactose, 2′-FL, 2′-FL/LNnT, or HMO6. For each feeding cycle, 140 mL of baby M-SHIME^®^ feed at a pH of 3 was added to the stomach followed by 60 mL of pancreatic juice (NaHCO_3_ 2.5 g/L, pancreatin 0.9 g/L, oxgall 4 g/L) after 1.5 h of incubation, as previously described [29]. 

The donor was selected based on the ability to ferment all the substrates and presenting a fermentation profile closest to the average response of the five donors. Inoculation of baby M-SHIME^®^ reactors was performed as previously described [30].

**Figure 1:**
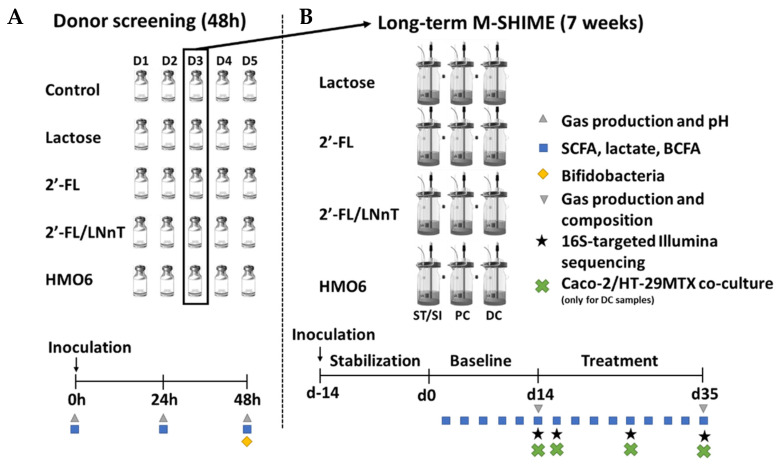
Schematic representation of the experimental setup: (**A**) Short-term incubations to screen fecal samples from five infant donors (48 h); (**B**) Long-term baby M-SHIME^®^ study using the selected fecal sample from the screening of a single infant. 2′-FL = 2′-fucosyllactose; LNnT = lacto-N-neotetraose, mixture of six human milk oligosaccharides [2′-FL, Lacto-N-tetraose (LNT), LNnT, difucosyl-lactose (LDFT), 3′SL, 6′SL (HMO6). D1-D5 = donors. SFCA = short-chain fatty acid; BCFA = branched-chain fatty acid; ST/SI = stomach/small intestine; PC = proximal colon; DC = distal colon; baby M-SHIME^®^ = mucosal simulator of the human intestinal microbial ecosystem.

The configuration of the baby M-SHIME^®^ used in this study is shown in Figure 1. The experimental timeline of the run consisted of a two-week stabilization period (day −14 to day 0), during which the fecal microbiota differentiated into communities representative of a specific colon region, followed by a two-week baseline period (day 0 to day 14) and a three-week treatment period (day 14 to day 35). Baseline values after 14 days of fecal inocula stabilization were obtained and referred to as the control condition.

### 2.4. Microbial Community Analysis by qPCR

Samples collected after 48 h during the short-term incubations were evaluated for the total amount of *Bifidobacterium* species by qPCR. DNA was isolated as described before [33] with minor modifications [34] from either 1 mL luminal samples or 0.25 g mucus samples. Subsequently, qPCR was performed using a QuantStudio 5 Real-Time PCR system (Applied Biosystems, Foster City, CA, USA). Each sample was run in technical triplicate and outliers with more than 1 C_T_ difference were omitted. The qPCRs were performed as described previously with the primers Bif243F (5′-TCGCGTCYGGTGTGAAAG-3′) and Bif243R (5′CCACATCCAGCRTCCAC-3′), which target the 16S rRNA gene [35]. Results are reported as log(16S rRNA gene copies/mL).

### 2.5. Microbial Community Analysis by 16S rRNA Gene Sequencing 

Microbial community composition was assessed before (day 14) and after treatment with 2′-FL, lactose, and HMO6 (day 16, day 26, and day 35). Samples were sent out to LGC Genomics GmbH (Berlin, Germany) for next-generation 16S rRNA gene amplicon sequencing of the V3–V4 region. Library preparation and sequencing were performed using an Illumina MiSeq platform with v3 chemistry. The 341F (5′-CCTACGGGNGGCWGCAG-3′) and 785R (5′-GACTACHVGGGTATCTAAKCC-3′) primers were used as previously described [36] with the reverse primer being adapted to increase coverage. Quality control PCR was conducted using Taq DNA Polymerase with the Fermentas PCR Kit according to the manufacturers’ instructions (Thermo Fisher Scientific, Waltham, MA, USA.). The DNA quality was verified by electrophoresis on a 2% (*w*/*v*) agarose gel for 30 min at 100 V. Bioinformatics analysis of amplicon data was performed as previously described [37].

#### Metabolic Analysis

pH measurements were performed using a Senseline pH meter F410 (ProSense, Oosterhout, The Netherlands). The gas formation was measured using a needle-equipped pressure meter (Hand-held pressure indicator CPH6200; Wika, Echt, The Netherlands). The gas-phase composition was analyzed using a compact GC (Global Analyser Solutions, Breda, The Netherlands), equipped with a Molsieve 5A pre-column and Porabond column (for CH_4_, O_2_, H_2_, N_2_), an Rt-Q-bond pre-column and column (for CO_2_, N_2_O, and H_2_S), and a thermal conductivity detector. The parameters used to evaluate the activity of the gut microbiota were monitored three times per week during the baseline (day 3/5/7/10/12/14) and treatment period (day 16/19/21/23/26/28/30/33/35). SCFA (acetate, propionate, and butyrate) and BCFA (isobutyrate, isovalerate, and isocaproate) were determined as previously described [38]. Lactate production was assessed with an enzymatic kit (R-Biopharm, Darmstadt, Germany), according to the manufacturer’s instructions.

### 2.6. Cell Lines

The human colorectal adenocarcinoma cell line Caco-2 (HTB-37) was obtained from the American Type Culture Collection (ATCC, Manassas, VA, USA) in passage 21 and used in experiments in passages 23 to 33. The human colon adenocarcinoma cell line HT29 (HTB-38; ATCC) previously adapted with methotrexate (MTX) was obtained from the European Collection of Authenticated Cell Cultures (ECACC, Salisbury, UK) at passage 51 and used in experiments at passages 23 to 33. Both cell lines were separately maintained in 75-cm^2^ tissue culture flasks (Thermo Fischer Scientific, Waltham, MA, USA.) in a humidified atmosphere of 37 °C and 10% CO_2_, 95% air/water-saturated atmosphere.

### 2.7. Cell Culture Procedures and Treatments

Both Caco-2 and HT29-MTX cell lines were maintained in Dulbecco’s Minimal Essential Media (DMEM; 11965092, Gibco™, Thermo Fischer Scientific, Waltham, MA, USA.) supplemented with 10% (*v*/*v*) heat-inactivated FBS (10270-106, Gibco™, Thermo Fischer Scientific, Waltham, MA, USA.) and 1% (*v*/*v*) Penicillin-Streptomycin solution (P4333, Sigma). The growth medium was replaced at a minimum of twice per week. Cell lines were subcultured weekly at preconfluent densities with 0.5 g porcine trypsin and 0.2 g EDTA (T3924, Sigma-Aldrich, Saint-Louis, MO, USA.).

For the experiments, Caco-2 and HT29-MTX cells were stained with trypan blue (T8154, Sigma-Aldrich, Saint-Louis, MO, USA), counted, resuspended in a complete growth medium at ratios of 76:24 to simulate the large intestine, and seeded at a density of 6 × 10^4^ cells per cm^2^ in Transwell™ Polycarbonate semi-permeable membrane of 0.4 µM pore size and 1.12 cm^2^ surface area (3460, Corning Life Sciences, Tewksbury, MA, USA.). Confluency and integrity of the Caco-2:HT29-MTX culture were evaluated by manually measuring the transepithelial electrical resistance every week using a Millicell™ ERS-2 Voltohmmeter (MERS00002, Merk-Millipore, Burlington, MA, USA.). Cells were used for experiments 21-days post-seeding.

On the day of the experiment, cell growth media were replaced by a fresh medium with all supplements but without phenol red. Fermented media from baby microbiota fed with lactose, 2′-FL, 2′-FL/LNnT, or HMO6 were added to the apical compartment of the transwell at a concentration of 20% *v*/*v*. Fresh, unfermented culture SHIME^®^ media was used as a control. After 36 h, epithelial barrier dysfunction was induced in cells exposed to lactose and HMO fermented media and in half of the unfermented media monolayers (Media +) by adding TNF-α (2.5 ng/mL) and IFN-γ (10 ng/mL) at the basolateral compartment of the Transwell™ for an additional 48 h. The remaining half of the unfermented media cells did not receive the cytokine challenge (Media-) and was used as an intact barrier reference group. 

### 2.8. Epithelial Permeability Assessment

Permeability was assessed using two readouts: trans-epithelial resistance (TEER) and translocation of FITC-labeled dextran (FD4; 4000 Da, Sigma-Aldrich, Saint-Louis, MO, USA) from the apical to basolateral compartment of the Transwell™.

TEER was dynamically measured every 5–15 min by placing the Transwell™ seeded with Caco-2:HT29-MTX culture in a cellZscope machine (Nano Analytics) inside a humidified atmosphere of 37 °C and 10% CO_2_, 95% air/water-saturated atmosphere for the whole duration of the experiment. TEER was measured as Ω per cm2 and percent change in TEER (% TEER change) was calculated relative to the baseline value (TEER measurement prior to any treatment). The % TEER changes at (i) the end of the 36-h pre-challenge period and (ii) the end of the 48-h post-challenge period were computed and used for data analysis. 

FD4 translocation was measured at the end of the 48-h post-challenge period by adding a filter-sterilized solution of FD4 in the apical compartment of Transwell™ at a final concentration of 1 mg/mL. Basolateral samples were collected before and 90 min after the apical addition of FD4. FD4 translocation was measured by interpolating the fluorescent intensity in the samples against a standard curve and expressed as ng/mL. FD4 translocation was normalized relative to the value in Media +.

Permeability experiments were performed in duplicate or triplicate wells and conducted in a single experiment.

The analyzed endpoints were: (i) the TEER value at the end of the 36-h pre-challenge period, (ii) the TEER value at the end of the 48-h post-challenge period, and (iii) the FD4% translocation at the end of the 48-h post-challenge period. All endpoints are expressed relative to Media + value = 100%, with standardization performed per plate.

### 2.9. Data Analyses

Descriptive statistics—namely arithmetic mean and standard error of the mean (SE) for all treatment groups—were used to summarize all outcomes of both experiments. In the case of bifidobacteria, data were priori log-transformed because of their log-normal distribution. The significance level of all statistical tests was set to 5%.

In the short-term incubation experiment, the significance of differences between treatments was assessed for all outcomes on the change between 0 h and 48 h, using a mixed-model with treatment as a fixed and donor as a random effect. A paired t-test was used for post-hoc pairwise comparisons of treatments, with a Bonferroni correction to account for multiplicity. Multivariate differences between treatments were further mapped using principal component analysis performed on normalized mean data of all outcomes.

In the long-term baby M-SHIME^®^ experiment, 16S rRNA sequencing data was initially processed as described in De Paepe et al. [37]. Briefly, the mothur software package (v.1.33.3) was used to assemble forward and reverse reads and contigs with a length between 441 and 467 bases aligned to the mothur formatted silva_seed release 119 alignment database. After removing non-aligning sequences as well as sequences containing homopolymer stretches of more than 12 bases, sequences were preclustered allowing up to four differences. UCHIME was applied to remove chimera and, subsequently, sequences were classified by means of a naive Bayesian classifier, against the RDP 16S rRNA gene training set, version 14 with an 80% cutoff for the pseudo-bootstrap confidence score. Only bacterial sequences were retained. A total of 1,378,852 withheld sequences were binned into Operational Taxonomic Units (OTUs) within each order identified by the preceding classification step. An OTU is defined in this manuscript as a collection of sequences with a length between 430 and 465 nucleotides that are found to be more than 97% similar to one another in the V3-V4 region of their 16S rRNA gene after applying hierarchical clustering. Finally, taxonomy assignment was obtained according to the RDP version 14 and silva.nr_v119 database. Data at phylum, family, and genus levels were processed using Calyspo software version 8.84, removing samples with less than 0.01% of abundance and applying a total sum normalization. Discriminant Analysis of Principal Components and an Adonist test based on Bray–Curtis distance were used to evaluate the differences between treatment groups, while the Linear discriminant analysis Effect Size was used to determine specific features at family and genus levels differentially enriched by treatments. Significant differences between treatments considering different time points were evaluated by Mixed Effect Linear Regression models. Mean relative distributions of microbiota at family and phylum levels were represented using stacked area charts for days 14, 16, 26, and 35.

For epithelial permeability outcomes, treatments were compared for change between baseline period (day 14) and treatment period (days 16, 26, and 35), using a one-way repeated measures analysis of variance. For all other outcomes, treatments were compared for change between baseline period (average of days 3 to 14) and treatment period (days 16 to 35), using a one-way repeated measures analysis of variance. A paired t-test was used for all post-hoc comparisons, with a Bonferroni correction to account for multiplicity. Multivariate trajectories of treatments over time (day 14, 16, 26, 35) were further mapped using principal component analysis performed on normalized mean data of all outcomes.

Statistical analysis was performed using GraphPad Prism version 8.2.0 (435) for Windows (GraphPad Software, San Diego, CA, USA). Principal Component Analysis (PCA) and Principal Component Regression plots were conducted with R 3.5.0.

### 2.10. Ethics 

Fecal samples of the five infants were collected according to the ethical approval of the University Hospital Ghent (reference number B670201836585). Informed consent of legal representatives was obtained after providing them with detailed information about the project and the use of the samples. 

## 3. Results

### 3.1. Different Mixtures of HMOs Induce a Fast Bifidogenic Environment and Metabolic Shift

The effect of lactose and HMO supplementation on bifidogenic potential and microbial metabolic activity was assessed by measuring copies of bifidobacteria by qPCR, pH changes, gas production, SCFA, branched short-chain fatty acids (BCFA), and lactate and ammonia levels at different time points (6 h, 24 h, and 48 h) during the short-term colonic incubations using fecal inocula. Five donors were included to assess inter-individual differences. Based on this pre-screening experiment, one donor was selected for a long-term study performed using the baby M-SHIME^®^ [29].

**Figure 2:**
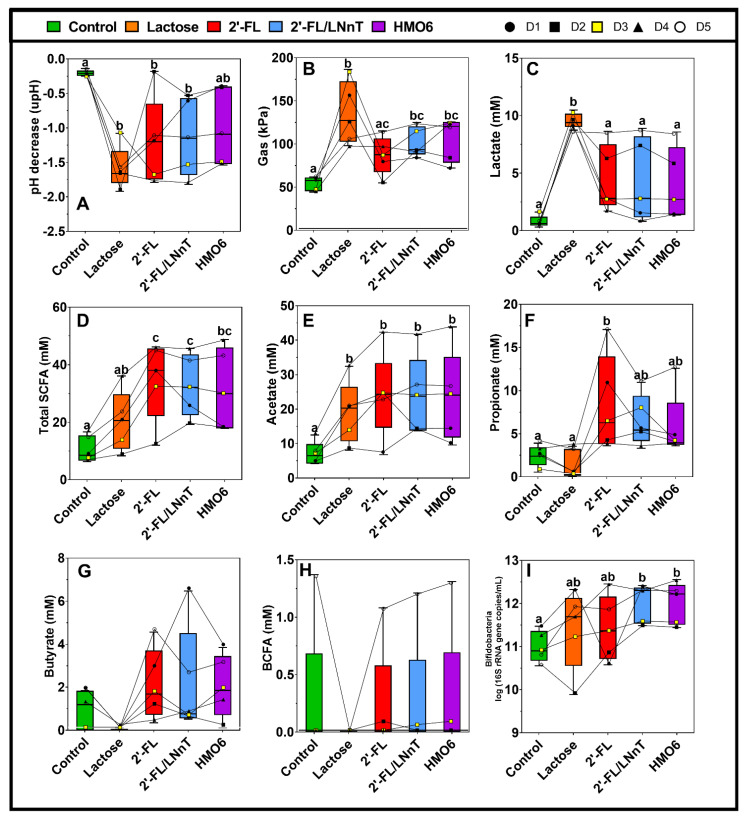
Effect of lactose, 2′-FL, 2′-FL/LNnT, or HMO6 on bacterial metabolic activity and bifidobacteria levels. (**A**) pH, (**B**) gas production, (**C**) lactate, (**D**) total SCFA, (**E**) acetate, (**F**) propionate, (**G**) butyrate, (**H**) BCFA, and (**I**) bifidobacteria levels during short-term fecal batch incubations (0–48 h), upon treatment with lactose, 2′-FL, 2′-FL/LNnT or HMO6. Box plots represent median and interquartile range (n = 5 donors, N = 3). Each line shows the effect of each treatment for one donor. Significant differences between treatments (one-way ANOVA with Bonferroni correction for all metabolic markers except BCFA which was analyzed with Friedman test with Dunn’s correction, due to normality assumption violation) are indicated with different letters (a, b, c; *p* < 0.05). Treatments sharing at least one letter are not significantly different.

In general, all treatments induced a decrease in pH compared to the control, with the highest effect observed for lactose and the lowest for HMO6 (Figure 2A, Table S1). 

Lactose induced a significant increase in the production of gas (Figure 2B, Table S1) and lactate (Figure 2C, Table S1) compared to the control condition. HMOs also increased gas production but at lower levels than lactose, and the effect of 2′-FL was not significant compared to the control (Figure 2B). In contrast, HMOs did not show a significant increase in lactate production (Figure 2C, Table S1).

All HMOs but not lactose significantly increased total SCFA compared to the control (Figure 2D, Table S1). Specifically, acetate production was increased by all the treatments compared to the control (Figure 2E), while only 2′-FL induced a significant propionate production (Figure 2F). Butyrate (Figure 2G, Table S1) and BCFA (Figure 2H, Table S1) production were not significantly affected by any treatment.

In addition, only 2′-FL/LNnT and HMO6 increased bifidobacteria levels as compared to the control (Figure 2I).

**Figure 3:**
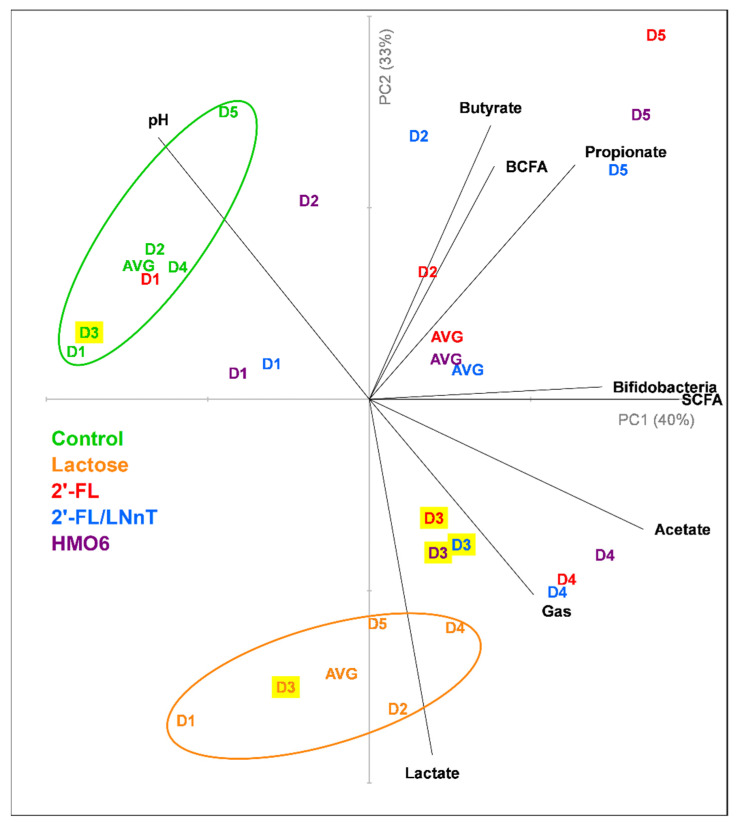
Principal component analysis plots representing metabolic data (pH, Gas, Acetate, Propionate, Butyrate, Lactate, BCFA, and total SCFA) and bifidobacteria levels obtained for control, lactose, 2′-FL, 2′-FL/LNnT, and HMO6 after 48 h of incubation with fecal microbiota from five breastfed infant donors. BCFA = branched-chain fatty acid; SCFA= short-chain fatty acid; 2′-FL = 2′-fucosyllactose; LNnT = lacto-N-neotetraose; D = donor; PC = principal component; AVG = average. Ellipses represent a 95% confidence interval and are only included for the control and lactose, due to the widespread and donor-dependent response for 2′-FL, 2′-FL/LNnt, and HMO6.

A principal component analysis plot representing the metabolic data and bifidogenic levels was generated in order to better visualize the overall treatment effect for each donor (Figure 3). It revealed that lactose clustered separately from the other treatments and the control. 2′-FL, 2′-FL/LNnT, and HMO6 showed a similar metabolic profile, especially in donors 3, 4, and 5. Donors 1 and 2 responded to the HMO treatments to a lesser extent than donors 3–5, clustering closer to the control condition. Donor 3 was selected for long-term studies as it closely resembled the average data of the five donors.

#### HMOs Promote SCFAs Production without a Gas Increase 

The effect of long-term and repeated doses of lactose and HMOs on the baby M-SHIME^®^ microbial activity was assessed with donor 3 inoculum by quantifying microbial metabolites at different time points during the baseline and treatment periods, while gas production and composition were evaluated by an off-line method at the end of the treatment period (day 35) of the baby M-SHIME^®^ run. Unless otherwise stated, results from the distal colon compartment only are described. The rationale for selecting the distal colon is based on longer transit times [39] and potentially more effects of the administered products on resident gut microbiota as compared to the proximal colon. The design of the experiment is summarized in Figure 1. 

**Figure 4:**
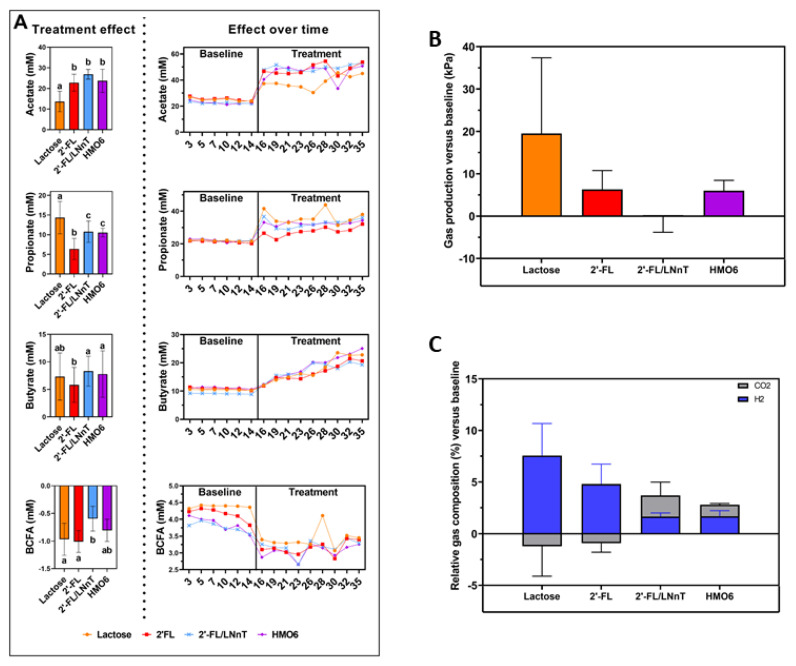
Effect of long-term treatment with lactose, 2′-FL, 2′-FL/LNnT, and HMO6 on bacterial metabolic activity. (**A**) Effect of lactose, 2′-FL, 2′-FL/LNnT, and HMO6 on acetate, propionate, butyrate, and BCFA levels (mM) in the distal colon. Bar plots in the left panel represent the mean ± standard deviation (SD) of the values at different time points (n = 9), after correction by the mean values for the baseline (n = 6 time points) for each reactor. This helps to observe the overall effect of each treatment after considering the effect of the baseline period. Time-course graphs in the right panel represent single measures of different metabolites at different time points. (**B**) Effect of lactose, 2′-FL, 2′-FL/LNnT, and HMO6 on gas production measured at day 35 of treatment and corrected by baseline values (n = 3). (**C**) Effect of lactose, 2′-FL, 2′-FL/LNnT, and HMO6 on the percentage of CO_2_ and H_2_ production measured at day 35 of treatment and corrected by baseline values (n = 3). Significant differences between treatments (one-way ANOVA with Bonferroni correction) are indicated with different letters (a, b, c; *p* < 0.05). Treatments sharing at least one letter are not significantly different.

In general, lactose, 2′-FL, 2′-FL/LNnT, and HMO6 increased short-chain fatty acid production (Figure 4A). Higher levels of acetate were observed from day two after treatment with lactose and HMOs, with significantly higher levels for HMOs compared to lactose (Figure 4A). Propionate also increased after two days of treatment, with more pronounced effects observed for lactose than for HMOs (Figure 4A). Butyrate similarly increased from baseline levels with different treatments, however, at the end of the treatment (day 35), the highest levels were found in the HMO6-treated reactors, compared to lactose, 2′-FL, and 2′-FL/LNnT (Figure 4A). In contrast, BCFA levels during the treatment period were significantly lower (*p* < 0.01) for lactose and 2′-FL compared to baseline levels (Figure 4A). Similar trends were observed in the proximal colon (Figure S1). 

When evaluating gas production as a marker of bacterial fermentation, lactose did not induce a significant increase compared to the other treatments (Figure 4B). Changes in gas composition were not significant (Figure 4C).

### 3.2. 2′-FL, 2′-FL/LNnT, and HMO6 Induce Bifidobacteriaceae Family in a Product-Dependent Way

The overall effect of lactose and HMOs on microbial modulation in the luminal and mucosal compartments of the baby M-SHIME^®^ was evaluated by 16S rRNA gene sequencing. Similar trends were observed in the proximal and distal compartments. Specific treatment, time, and compartment effect are described in detail in the following paragraphs.

Overall, none of the treatments significantly affected diversity or evenness in either the luminal or the mucosal compartment (Figure S2).

**Figure 5:**
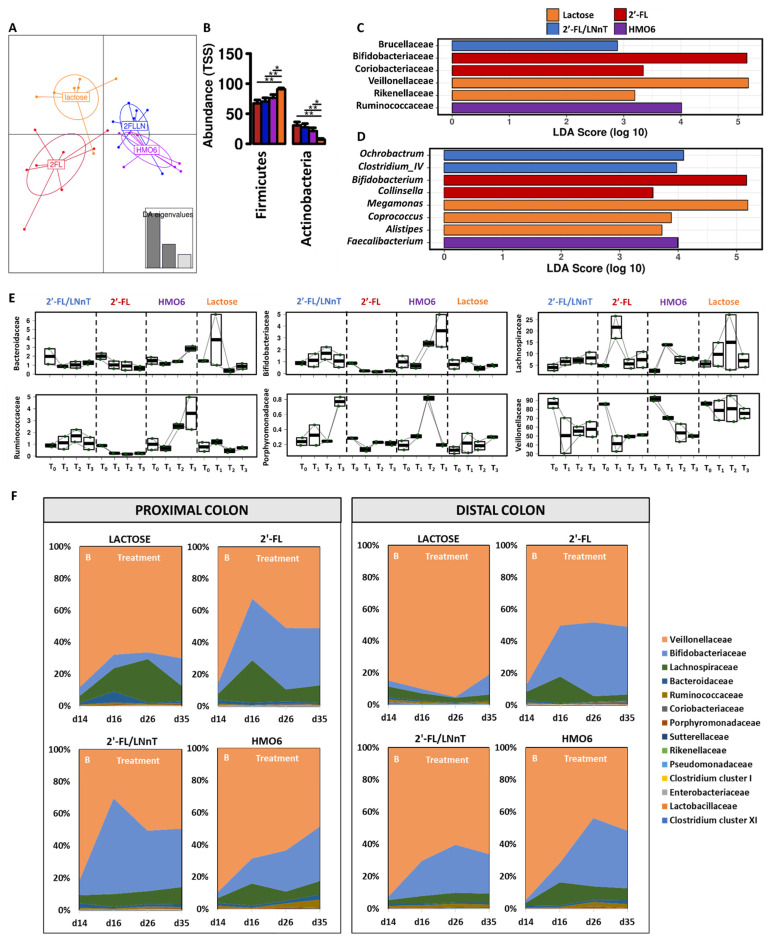
Effect of lactose, 2′-FL, 2′-FL/LNnT, and HMO6 on baby M-SHIME^®^ microbiota structure. (**A**) Discriminant Analysis of Principal Components (DAPC) of the luminal PC and DC at the family level. (**B**) Bar plot and ANOVA analysis at the phylum level. Linear Discriminant Analysis Effect Size (LEfSe) at the family (**C**) and genus (**D**) level of the luminal compartment. (**E**) Selected features significantly affected by different treatments at the genus level using a mixed effect model. (**F**) Proportional abundance at the family level (%) of a breastfed infant microbiota before (day 14) and after treatment with lactose, 2′-FL, 2′-FL/LNnT or HMO6 in the luminal proximal (PC) and distal colon (DC) of the baby M-SHIME^®^. B: Baseline. T_0_ = start of the experiment, T_1_ = 7 days, T_2_ = 14 days, T_3_ = 21 days.

Using a Discriminant Analysis of Principal Components (DAPC), luminal samples from the proximal colon (PC) and distal colon (DC) reactors treated with lactose or with 2′-FL were grouped separately from the other two treatments, which clustered together (*p* = 0.02, Adonis based on Bray–Curtis distances) (Figure 5A). 

Specifically, 2′-FL, 2′-FL/LNnT, and HMO6 increased the relative abundance of Actinobacteria while reducing Firmicutes, as compared to lactose (Figure 5B). At the family and genus level, a linear discriminant effect size analysis (LEfSe) showed that lactose increased the level in *Rikenellaceae* (*Alistipes*) and *Veillonellaceae* (*Megamonas*) families. HMOs had specific effects on different gut microbiota members, with an increased level in *Bifidobacteriaceae* (*Bifidobacterium*) and *Coriobacteriaceae* (*Collinsella*) by 2′-FL, and *Brucellaceae* (*Ochrobactrum*) by 2′-FL/LNnT. *Ruminococcaceae* (*Faecalibacterium*) family relative abundance was increased in HMO6 treated-reactors (Figure 5C,D). 

When considering the effect of time on modulation of the microbial community, we observed a consistent effect of HMOs reducing the *Veillonellaceae* family (*p* < 0.001, Figure 5E,F, Table S2), especially in the DC HMO6-treated reactor across the three weeks of treatment. Reductions in the *Veillonellaceae* family (*p* < 0.001) were also observed during the first week of treatment with 2′-FL and 2′-FL/LNnT but were slightly recovered at the end of the treatment period, whereas the reduction was minimal with lactose (Figure 5E,F, Table S2). The *Lachnospiraceae* family level was increased by different treatments (*p* < 0.001) and intermediate increases were maintained in time using the HMOs mixtures (2′-FL/LNnT and HMO6), while 2′-FL showed the highest increase during the first week of treatment, dropping to basal levels in the next time points, with a similar tendency observed with lactose (Figure 5E,F, Table S2). Changes in *Bifidobacteriaceae* and *Ruminococcaceae* abundance were specific for HMO6, with increases observed from the second week of treatment and maintained until the end of the assay (Figure 5E,F, Table S2). *Bacteroidaceae* changes were mild, with an overall reduction for 2′-FL and 2′-Fl/LNnT (Figure 5E,F, Table S2). This reduction was also observed for lactose, despite an increase during the first week of treatment. HMO6 was, however, the only treatment that showed an increase in *Bacteroidaceae* levels at the end of the treatment period. *Porphyromonadaceae* accounted for only a fraction of the total families, with mild changes with 2′-FL (reduction) and with lactose (increase) (Figure 5E,F, Table S2). An intermediate *Porphyromonadaceae* increase level was observed during the second week with HMO6 that resumed to basal level at the end of treatment, while 2′-FL/LNnT was the only treatment that showed an increase during the last week only.

At the OTU level, a LEfSe analysis showed specific OTUs enrichment in the luminal compartment depending on the treatment (Figure S3, Tables S3 and S4). In the following paragraph, the most closely related bacterial species for each OTU is indicated in between brackets. Lactose-treated reactors were enriched in OTU1 (*Megamonas* sp.), OTU2 (*B. longum*), and OTU31 (*Allistipes finegoldi*). 2′-FL treatment was associated with high levels of OTU3 (*B. adolescentis*), OTU29 (*Collinsella aerofaciens*), and OTU38 (*Sutterella* sp.). OTU36 (*Clostridium* sp.) was enriched specifically in 2′-FL/LNnT reactors, while HMO6 primarily and consistently enriched OTU20 (*Faecalibacterium prausnitzii*) and OTU35 (*B. dentium*) levels alongside other minor OTUs such as OTU53 (Uncultured bacterium), OTU56 (*Bacteroides vulgatus*), and OTU59 (*Sutterella* sp.) (Figure S3, Tables S3 and S4). 

Further, several low-abundant *Bifidobacteriaceae* OTUs were differentially enriched upon HMO treatment. HMO6 (day 26) and 2′-FL/LNnT (day 16) caused an increase in the level of the most OTUs from the *Bifidobacteriaceae* family, including OTUs 2, 3, 9, 35, and 44, related to *B.longum*, *adolescentis*, *bifidum,* and *dentium*, respectively (Tables S3 and S4). OTU2 (related to *B. longum*) was initially (day 16) stimulated by 2′-FL/LNnT especially. OTU9 (related to *B. bifidum*) and OTU 35 (related to *B. dentium*) gradually increased in abundance with 2′-FL and HMO6 treatments. In contrast, OTU9 and OTU44 (both associated with *B. bifidum*) were only initially enriched upon 2′-FL/LNnT treatment (day 16) (Tables S3 and S4). 

In the mucosal compartment, the Adonis test was not significant (*p* = 0.593, family level), but particular changes at family, genus, and OTU levels were observed (Figure S4A). At the family level, a LEfSe analysis showed that 2′-FL treatment induced an enrichment in *Rikenellaceae* and *Coriobacteriaceae*, while 2′-FL/LNnT-treated reactors were enriched in *Lactobacillaceae* (Figure S4B). Increased *Bifidobacteriaceae* and *Lachnospiraceae,* as well as decreases in Veillonellaceae, were also observed (Table S5), following a similar observation as in the luminal compartment. 

*Megamonas* abundance was reduced at the genus level with a more immediate effect observed for HMOs compared to lactose. Especially, 2′-FL/LNnT and HMO6 had a consistent and strong effect after the second week of treatment (Figure S4E). *Bifidobacterium* level increases were observed for all the treatments; however, lactose showed a delayed effect, only observed at the end of the treatment (Figure S4E). Only lactose increased transiently *Clostridium* cluster XIV abundances, while *Roseburia* was mainly stimulated by HMO6 (Figure S4E). Overall, *Ruminococus* levels were increased by different treatments, especially in the second week of treatment, with a return to basal level by the end of the treatment with HMO6 and 2′-FL (Figure S4E). 

At the OTU level, a LEfSe analysis showed enrichment in OTU2 (*B. longum*) and OTU7 (*Clostridium clostridioforme*) in lactose, OTU29 (*Collinsella aerofaciens*) and OTU31 (*Allistipes finegoldi*) in 2′-FL, OTU9 (*B. bifidum*) and OTU36 (*Clostridium* sp.) in 2′-FL/LNnT, and OTU10 (*Roseburia inulinivorans*) and OTU63 (*Ruminococcus lactaris*) in HMO6-treated reactors (Figure S5A). OTU1 (*Megamonas* sp.) relative abundance was also reduced by all the treatments in the mucosal compartment (*p* = 0.002). OTU2 (*B. longum*) levels were decreased by different HMOs, while OTU3 (*B. adolescentis*) relative abundance was highly induced by HMO6 from the first week. The same effect was observed for 2′-FL/LNnT in the second week and only at the end of the treatment for lactose (Figure S5, Tables S6 and S7) (*p* < 0.001). OTU7 (*Clostridium clostridioforme*) was specifically enriched by lactose treatment during the second week, an effect not observed for the HMOs. Similarly, only 2′-FL increased the relative abundance of OTU12 (*Sporanaerobacter acetigenes*), especially at the end of the treatment period. In general, OTU8 (*Ruminococcus torques*/*faecis*), OTU9 (*B. bifidum*), OTU10 (*R. inulinivorans*), and OTU44 (*B. bifidum*) were enriched by all the treatments with a specific product time-trend (Figure S5, Tables S6 and S7). In general, HMO6 showed the fastest and highest bifidogenic potential. 

### 3.3. HMOs Fermentation Products but Not Lactose Protect the Intestinal Barrier from a Pro-Inflammatory Challenge

**Figure 6:**
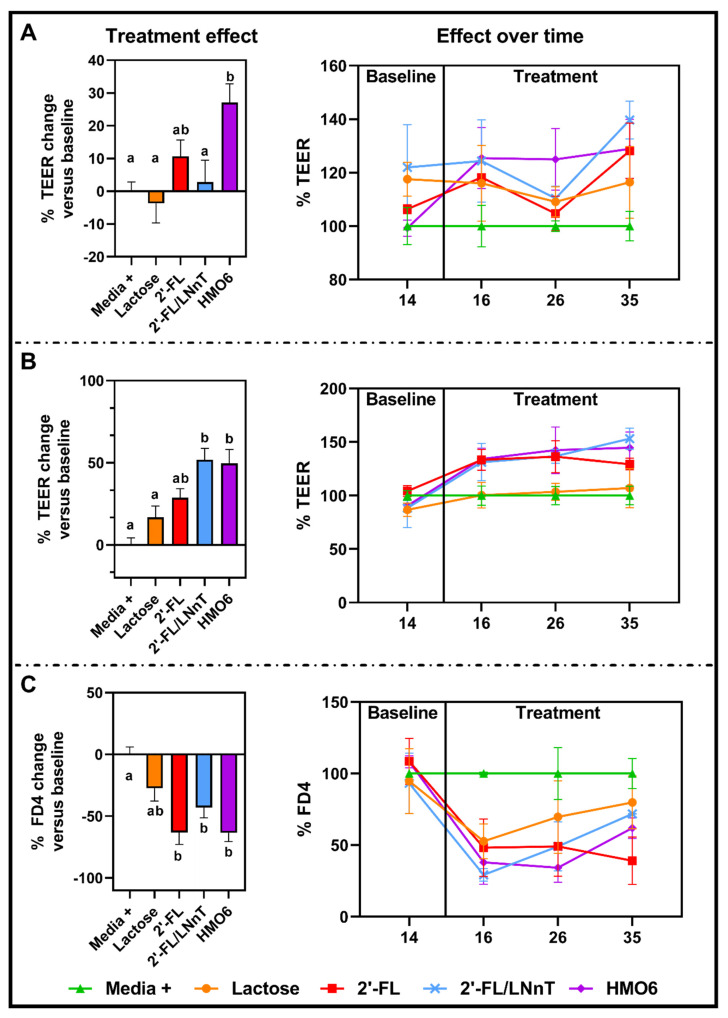
Effect of lactose, 2′-FL, 2′-FL/LNnT, and HMO6 fermentation products on intestinal barrier function in vitro. Percentage of TEER values with respect to baseline (day 14) supernatant values in intact (**A**) or IFNγ/TNFα-challenged (**B**) Caco-2/HT29-MTX monolayers exposed to baby M-SHIME^®^ supernatants (36 h) from 2 (day 16), 12 (day 26), and 21 (day 35) days of treatment. (**C**) Percentage of FD4 transport to the basolateral compartment with respect to the baseline (day 14) supernatant values in IFNγ/TNFα-challenged Caco-2/HT29-MTX monolayers exposed to baby M-SHIME^®^ supernatants (36 h) from 2 (day 16), 12 (day 26), and 21 (day 35) days of treatment. Bar plots in the left panel represent the mean ± standard error of the mean (SEM) values at different time points (n = 9) of each treatment. Time-course graphs in the right panel represent measures (mean ± SEM, n = 3) at different time points, and data are expressed relative to Media+ (100%) at each time point. Significant differences between treatments (one-way ANOVA with Bonferroni correction) are indicated with different letters (a, b; *p* < 0.05). Treatments sharing at least one letter are not significantly different.

The effect of filter-sterilized baby M-SHIME^®^ supernatants from the distal colon obtained on days 14 (baseline), 16 (two days of treatment), 26 (12 days of treatment), and 35 (21 days of treatment) on the intestinal barrier was evaluated in a co-culture of Caco-2/HT29-MTX in intact conditions (Figure 6A) and after a pro-inflammatory challenge (Figure 6B,C). 

Before the pro-inflammatory challenge, the mean trans-epithelial electrical resistance (TEER) values in the cells incubated with the different HMO6 supernatants were significantly higher than in control cells (Figure 6A and Figure S7). In contrast, the mean TEER values obtained with lactose, 2′-FL, and 2′-FL/LNnT supernatants from the different treatment times did not significantly differ from the control condition.

Challenging the cell monolayers with a pro-inflammatory stimulus significantly reduced the TEER (54.6 ± 3.5%) in the control monolayers (Media +), showing damage to the intestinal barrier as compared to the non-challenged condition (Media-, 102.8 ± 6.4%) (Figure S7). Incubation with supernatants from baby M-SHIME^®^ dosed with 2′-FL/LNnT and HMO6 significantly prevented the TEER drop (Figure 6B). 2′-FL did not significantly prevent the TEER drop, yet showed higher protection than lactose, and the effect was not significantly different than the other two HMOs treatments.

The protective effect of HMOs on barrier function against pro-inflammatory damage was confirmed by the paracellular transport of the FD4 assay. Fermented media from baby microbiota fed with 2′-FL, 2′-FL-LNnT, and HMO6, but not lactose, significantly decreased the translocation of FD4 to the basolateral compartment compared to the control (Media +) (Figure 6C and Figure S7).

### 3.4. HMOs and Lactose Have Different Effects on Intestinal Homeostasis

**Figure 7:**
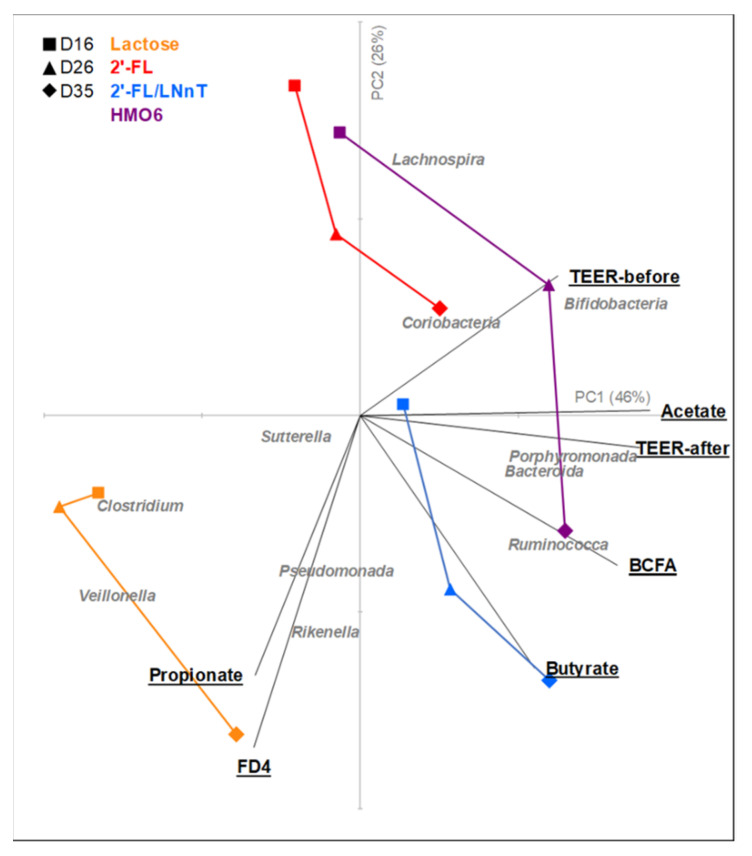
Principal component regression plot representing distal colon microbial activity data, bifidobacteria levels, and intestinal barrier function (TEER and FD4 translocation before and after pro-inflammatory challenge) obtained for 2 (day 16), 12 (day 26), and 21 (day 35) days of treatment with blank control, lactose, 2′-FL, 2′-FL/LNnT, or HMO6. Data were plotted on the first two principal components, contributing to 46% (PCA1) and 26% (PCA2). Lines connect different time points belonging to the same treatment group.

To visualize the overall impact of the treatments on intestinal ecology, the microbiota and intestinal barrier read-outs were combined in a Principal Component Regression analysis. PC1 and PC2 explained 46% and 26% of the variance with lactose affecting the intestinal ecosystem differently than the HMOs. The time of the treatment also had an impact on the modulation of the microbiota activity, structure, and host response, with a similar pattern for 2′-FL and HMO6 after 2 days of treatment (day 16), but with a more similar evolution of HMOs mixtures (2′-FL/LNnT and HMO6) towards the end of the treatment (day 35). Veillonella and propionate clustered together whereas acetate, bifidobacteria, and TEER are closer to PC1 and in between them. HMOs differentially modulated the microbial and host ecosystem with a higher effect than lactose observed in TEER, butyrate, ruminococca, and lachnospira modulation, especially by 2′-FL/LNnT and HMO6 (Figure 7). Similar trends were observed when only metabolic and compositional microbial data were included in the Principal Component Regression analysis of the proximal and distal colon (Figure S6).

## 4. Discussion

The present study evaluated the short- and long-term effects of lactose, 2′-FL, 2′-FL/LNnT, and HMO6 on breastfed infant gut microbiota and the role of fermentation metabolites derived from these products on intestinal barrier integrity in vitro. The short-term assays included screening fecal inoculum from five breastfed infants to encounter inter-individual differences. The results showed a consistent fermentation of all the products with donor-specific differences more evident for the HMOs, while lactose had a more similar profile to the control. Common features of bacterial fermentation of the different HMOs and lactose were (i) the increase in acetate and lactate production and (ii) a consequent pH reduction. In contrast, gas production was mainly observed in lactose-treated reactors and less so with HMO treatments. Excessive intestinal gas is frequently associated with abdominal distress [40]. Low gas levels in HMO-supplemented reactors may suggest a lower risk of bowel discomfort and represent a benefit for the infant receiving HMOs and not just lactose.

2′-FL, 2′-FL/LNnT, and HMO6 had a strong and immediate bifidogenic effect which was associated with a quick and sustained increase in acetate levels. These results are aligned with previous in vitro and human intervention studies showing the promotion of bifidobacteria upon administration of 2′-FL alone or in combination with plant-derived oligosaccharides or with LNnT [25,26,41], whereas novel results are provided for HMO6. This rapid effect could be beneficial in infants, whose gastrointestinal transit time is short, therefore promoting a faster bifidobacterial growth that could promote a colonization advantage.

*Bifidobacterium* spp. are beneficial members of the gut microbiota and altered or delayed colonization is one of the most frequent features present in different diseases such as inflammatory conditions of the gut or immune-related disorders such as asthma [7]. The first months of life are especially sensitive to host-microbiota interaction. It is considered a window of opportunity in which the assembly of microbial communities within the gastrointestinal tract substantially affects the immune, endocrine, and metabolic homeostasis, with short and long-term effects on health [42]. In our study, the bifidogenic effect of HMOs was mainly due to stimulation of *B. adolescentis* and to a minor extent *B. dentium, bifidum,* and *longum*. Recently, Berger et al. (2020) reported that the fecal community of infants receiving HMOs shifted towards a higher abundance of bifidobacteria and clustered closer to breastfed infants [43]. The authors noted that differences at the species level were mainly due to the distinct abundance of *B. adolescentis* and *B. catenulatum* group [43], supporting the results in the baby M-SHIME^®^ presented here.

In addition to acetate and bifidobacteria abundances, HMOs and lactose also increased butyrate production during the long-term assay, potentially attributed to the enrichment in butyrate-producing species belonging to the *Lachnospiraceae* family. Remarkably, a mixture of HMOs (HMO6) simultaneously increased *B. adolescentis* and *F. prausnitzii*, the latter of which is a butyrate-producer associated with healthy gut microbiota [44]. Cross-feeding mechanisms in co-culture of *F. prausnitzii* and *B. adolescentis* have been previously described [45], and the widespread costless secretion of amino acids was suggested as a mechanistic explanation for the mutualistic link between *B. adolescentis* and *F. prausnitzii* [46]. Recurrent association of *F. prausnitzii* and *B. adolescentis* is found in literature and supported by the results presented here, indicating a mutual interplay between these two members of the human gut microbiota. Both commensal microbes have been linked to healthy gut ecosystems [47,48,49], supporting a beneficial link between microbiota, HMOs, and infant health.

The catabolism of HMOs by butyrate-producing Clostridiales has also been recently described. Concretely, *Roseburia* and *Eubacterium* spp. have the enzymatic machinery to degrade HMOs and grow on complex HMOs purified from mother’s milk and on defined HMO molecules [50]. In the present setup, the mucosal compartment was enriched in both *Bifidobacteriaceae* and Clostridiales family members, such as OTUs related to *Eubacterium* spp., *Ruminococcus* spp., *Roseburia* spp., or other butyrate-producing clostridia, especially after 2′-FL/LNnT and HMO6. Trophic interactions between *Bifidobacterium* spp. and *Eubacterium hallii* (reclassified as *Anaerobutyricum hallii* [51]) or *Anaerostipes cacae* have been reported in the presence of 2′-FL, resulting in the conversion of acetate and lactate to butyrate, and propionate production from 1,2-propanediol [52,53].

The relative abundance of *A. hallii* was increased in the mucosal compartment of the baby M-SHIME^®^, suggesting cross-feeding interactions between different infant microbial community members, especially at the mucosal interface.

In colicky infants, reduced bifidobacteria, *Eubacterium* spp., and *A. hallii* levels have been described, whereas in adult populations, reduced *C. clostridioforme*, the *E. rectale* group, *F. prausnitzii,* and bifidobacteria abundances in the mucosal tissue of inflammatory bowel disease patients have been previously reported [54]. Remarkably, all these microbial groups have been enriched with HMOs, suggesting a promising strategy to modulate gut microbiota in dysbiotic and/or inflammatory conditions through HMOs.

The colonic mucosal environment is in continuous cross-talk with the host, and it is a particular niche with differential characteristics from the luminal environment [55]. The ability of specific microorganisms to colonize and adhere to the mucus has been reported as a significant feature of immune modulation, intestinal maturation, and the competitive exclusion of pathogens [56]. Host-microbiota interplay is more intimate in the mucosal epithelium’s boundaries, where specific mucus-degrading communities cope with microaerophilic environments while producing metabolites that diffuse through the mucus layer and affect the host at local and systemic levels [57]. SCFAs reinforce the intestinal epithelial barrier, modulate energy homeostasis and metabolism, regulate appetite, balance immune responses, and are key molecules in gut-brain communication [58]. HMO supplementation increased acetate, butyrate, and propionate production, as well as SCFAs, with positive effects on host health. Lactose also increased SCFAs production, however, only positive effects on reinforcing the intestinal epithelial barrier were observed with HMOs. Remarkably, acetate levels were higher in HMOs compared to lactose, suggesting a role of acetate and potentially bifidobacteria for intestinal barrier protection as previously described [59].

In addition to SCFA, other beneficial metabolites may be produced in the presence of HMOs but not lactose, which complementary boost intestinal barrier homeostasis. Indeed, we observed that HMO, but not lactose fermented media, increased the tightness of the epithelial monolayer and protected it against the pro-inflammatory challenge. For HMO6, this effect was consistent for baby M-SHIME^®^ supernatants from the different treatment times. Previous studies have shown an increase in claudin-5 expression in human organoids derived from proximal, transverse, and distal colon biopsies exposed to microbial metabolites derived from microbial fermentation of 2′-FL [6]. To date, no information was previously available on the effect of fermented HMO complex mixtures on intestinal epithelial monolayer homeostasis and response to an inflammatory challenge.

Different mechanisms might be involved in the effect of baby M-SHIME^®^ supernatants on the intestinal barrier. For example, N-acetylglucosamine is one of the end products of lacto-N-biose degradation with protective effects on intestinal mucosal barrier dysfunction [60,61]. Other components of the bacterial wall such as lipopolysaccharide (LPS) from specific bacteria, pili proteins, or extracellular vesicles have regulatory effects on the intestinal homeostasis [62] and are likely to be present on the baby M-SHIME^®^ supernatants, with differential composition depending on the microbiota profile. The protective role of HMO metabolites or microbial products on inflammatory intestinal barrier disruption may promote intestinal homeostasis and reduce the contact of antigenic compounds with the internal environment, preventing further inflammation increase and chronicity. Non-degraded HMOs could also enable a protective effect on the intestinal barrier as previously observed in a Caco-2/HT29-MTX model simulating intestinal inflammation [28]. In the baby M-SHIME^®^ supernatants, intact HMOs were below detection limits, but the protective effect of HMOs on intestinal health in vivo could be synergic when both intact HMOs and HMO-fermented metabolites are present in the colon. This hypothesis would require further research.

Significant limitations of this study include: (i) the small number of donors that limited the extrapolation of results; (ii) the use of in vitro models lacking the complexity of the human physiology; (iii) the use of cell lines of carcinogenic origin that may influence the physiological response and translatability of the results, and (iv) microbiota analysis based on the taxonomic profile obtained via 16S rRNA gene sequencing, which has less resolution than other approaches such as complete shotgun metagenome sequencing or metabolomics.

Among the novelty and strengths of the study are: (i) the study of composition and metabolism of both luminal and mucosal microbiota; (ii) the assessment of the effect of bacterial metabolites on the host interface; (iii) the use of individual fecal inocula that retain the interindividual variability of the microbiota responses; (iv) the combination of physiologically relevant doses of HMOs; (v) the use of long-term fermentation with repeated treatment, and (vi) the use of defined yet complex HMOs mixtures that more closely mimic the human milk composition. Importantly, the use of the baby M-SHIME^®^ in vitro system allowed us to obtain mechanistic data on microbiota-host interplay, including the mucosal environment.

In summary, data obtained using the baby M-SHIME^®^ model showed that HMOs had a microbiota modulatory capacity and a fermentation profile consistently different from lactose, with significantly lower gas and higher acetate production induced by HMOs. All the tested products had a bifidogenic effect and increased SCFA levels, however, these effects were faster with HMOs than with lactose and only HMOs had a protective role against inflammatory intestinal barrier disruption. In addition, HMO6-derived metabolites were the only treatment that increased the barrier resistance before the inflammatory challenge. The bifidogenic effect of HMO6 was also especially high, suggesting a positive effect of complex HMO mixtures on adaptative mechanisms of *Bifidobacteriaceae* members to colonize and persist in the luminal and mucosal interface.

The combined HMO effect on supporting bifidobacteria colonization and promoting intestinal barrier function suggests that supplementation of infant formula with more complex HMO mixtures could help establish diverse bifidobacterial communities within the infant gut and prevent pro-inflammatory imbalances in the intestinal mucosa. Notably, these results provide additional insights into the mechanisms underlying the health benefits of human milk, especially in the presence of complex mixtures of HMOs. Overall, this study suggests that diversifying the nature of the HMOs supplementing the infant formula may contribute to the improvement of infant health and ultimately stress the importance of breastfeeding.

## Data Availability

The data that support the findings of this study are available upon request from the corresponding authors. 16S rRNA sequencing data is available at the NCBI repository under the bioproject ID PRJNA765654.

## Supplementary Materials

The following supporting information can be downloaded at: https://www.mdpi.com/article/10.3390/nu14122546/s1, Figure S1: Effect of lactose, 2′-FL, 2′-FL/LNnT, and HMO6 on acetate, propionate, butyrate and BCFA levels (mM) in the proximal colon. Figure S2: Effect of long-term exposure to lactose, 2′-FL, 2′-FL/LNnT, and HMO6 on microbial community diversity markers at luminal and mucosal compartments of the distal colon at the family level. Figure S3: Modulation of microbiota by lactose, 2′-FL, 2′-FL/LNnT, and HMO6 in the luminal compartment at OTU level. Figure S4: Effect of lactose, 2′-FL, 2′-FL/LNnT, and HMO6 gut microbial community at family and genus level in the mucosal compartment of the long-term M-SHIME^®^. Figure S5: Effect of lactose, 2′-FL, 2′-FL/LNnT, and HMO6 gut microbial community at OTU level in the mucosal compartment of the long-term M-SHIME^®^. Figure S6: Principal component regression plots of short-chain fatty acids and microbiota composition by 16S RNA seq considering proximal and distal colon compartment as different conditions or analyzed together. Figure S7: Time course effect of lactose, 2′-FL, 2′-FL/LNnT, and HMO6 fermentation products on intestinal barrier function in vitro. Table S1: Effect of lactose, 2′-FL, 2′-FL/LNnT, and HMO6 on bacterial activity markers and quantification of bifidobacteria by qPCR on five individual donors in short-term incubations in vitro. Table S2: Proportional microbial composition at the family level (%) as determined via 16S-targeted Illumina sequencing in the lumen of the proximal and distal colon compartments of the M-SHIME^®^ before (d14) and after (d16, d26, and d35) treatment with lactose, 2′-FL, 2′-FL/LNnT or HMO6. Table S3: Proportional microbial composition at the OTU level (%) as determined via 16S-targeted Illumina sequencing in the lumen of the proximal colon compartment of the M-SHIME^®^ before (d14) and after (d16, d26, and d35) treatment with lactose, 2′-FL, 2′-FL/LNnT or HMO6. Table S4: Proportional microbial composition at the OTU level (%) as determined via 16S-targeted Illumina sequencing in the lumen of the distal colon compartment of the M-SHIME^®^ before (d14) and after (d16, d26, and d35) treatment with lactose, 2′-FL, 2′-FL/LNnT or HMO6. Table S5: Proportional microbial composition at the family level (%) as determined via 16S-targeted Illumina sequencing in the mucus of the proximal and distal colon compartments of the M-SHIME^®^ before (d14) and after (d16, d26, and d35) treatment with lactose, 2′-FL, 2′-FL/LNnT or HMO6. Table S6: Proportional microbial composition at the OTU level (%) as determined via 16S-targeted Illumina sequencing in the mucus of the proximal colon compartment of the M-SHIME^®^ before (d14) and after (d16, d26, and d35) treatment with lactose, 2′-FL, 2′-FL/LNnT or HMO6. Table S7: Proportional microbial composition at the OTU level (%) as determined via 16S-targeted Illumina sequencing in the mucus of the distal colon compartments of the M-SHIME^®^ before (d14) and after (d16, d26, and d35) treatment with lactose, 2′-FL, 2′-FL/LNnT or HMO6.
