# Supplementary material for: Human Milk Oligosaccharides and Lactose Differentially Affect Infant Gut Microbiota and Intestinal Barrier In Vitro
Source: Nutrients. 2022 Jun 19;14(12):2546. doi: 10.3390/nu14122546 (PMC9227761; doi:10.3390/nu14122546)
Supplement: Supplementary file 1 [file nutrients-14-02546-s001.zip › nutrients-1775886-supplementary.pdf]

Human Milk Oligosaccharides and Lactose Differentially Affect Infant Gut Microbiota and Intestinal Barrier In Vitro

Jane Mea Natividad [1,†], Benoît Marsaux [2,3,*,†], Clara Lucia Garcia Rodenas [1], Andreas Rytz [1], Gies Vandevijver [2], Massimo Marzorati [2,3], Pieter Van den Abbeele [2], Marta Calatayud [2,3] and Florence Rochat [1]

[1] Nestlé Institute of Health Sciences, Société des Produits Nestlé S.A., Vers-Chez-Les-Blanc, CH-1000, Lausanne 26, 1000 Lausanne, Switzerland; janemeam.natividad@nestle.com (J.M.N.); clara_garcia@bluewin.ch (C.L.G.R.); andreas.rytz@rdls.nestle.com (A.R.); florence.rochat@rdls.nestle.com (F.R.)

[2] ProDigest BV, Technologiepark-Zwijnaarde 82, 9052 Ghent, Belgium; gies.vandevijver@prodigest.eu (G.V.); massimo.marzorati@prodigest.eu (M.M.); pieter.vandenabbeele@telenet.be (P.V.d.A.); marta.calatayudarroyo@gmail.com (M.C.)

[3] Center for Microbial Ecology and Technology (CMET), Faculty of Bioscience Engineering, Ghent University, Coupure Links 653, 9000 Ghent, Belgium

* Correspondence: benoit.marsaux@prodigest.eu

† These authors contributed equally to this work.

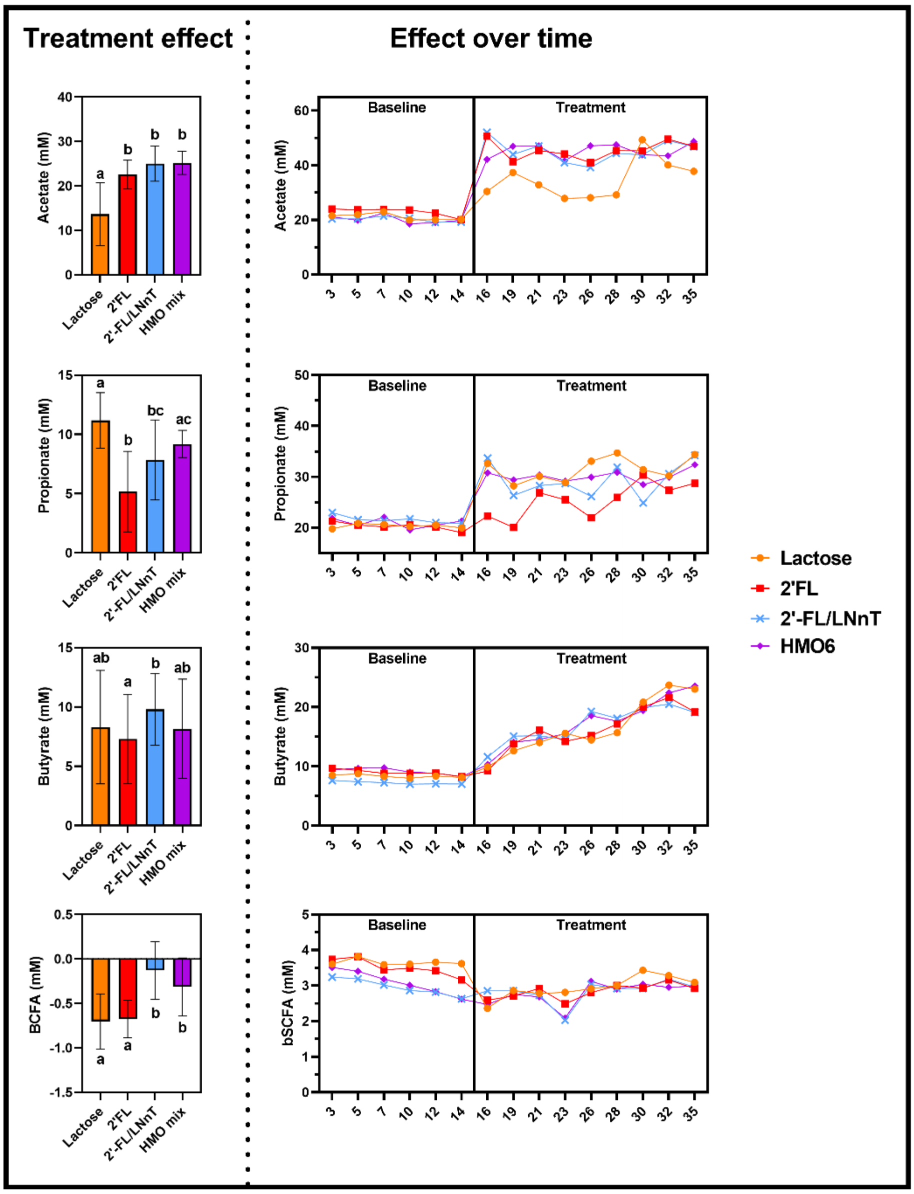

Figure S1: Effect of lactose, 2′-FL, 2′-FL/LNnT and HMO6 on acetate, propionate, butyrate and BCFA levels (mM) in the proximal colon. Bar plots in the left panel represent mean ± standard deviation of the values at different time points (n=9) corrected by the mean values for the baseline (n=6) for each reactor. Time-course graphs in the right panel represent single measures of different metabolites at different time points. Significant differences between treatments (one-way ANOVA with Bonferroni correction), are indicated with different letters (a, b, c; $p < 0.05$).

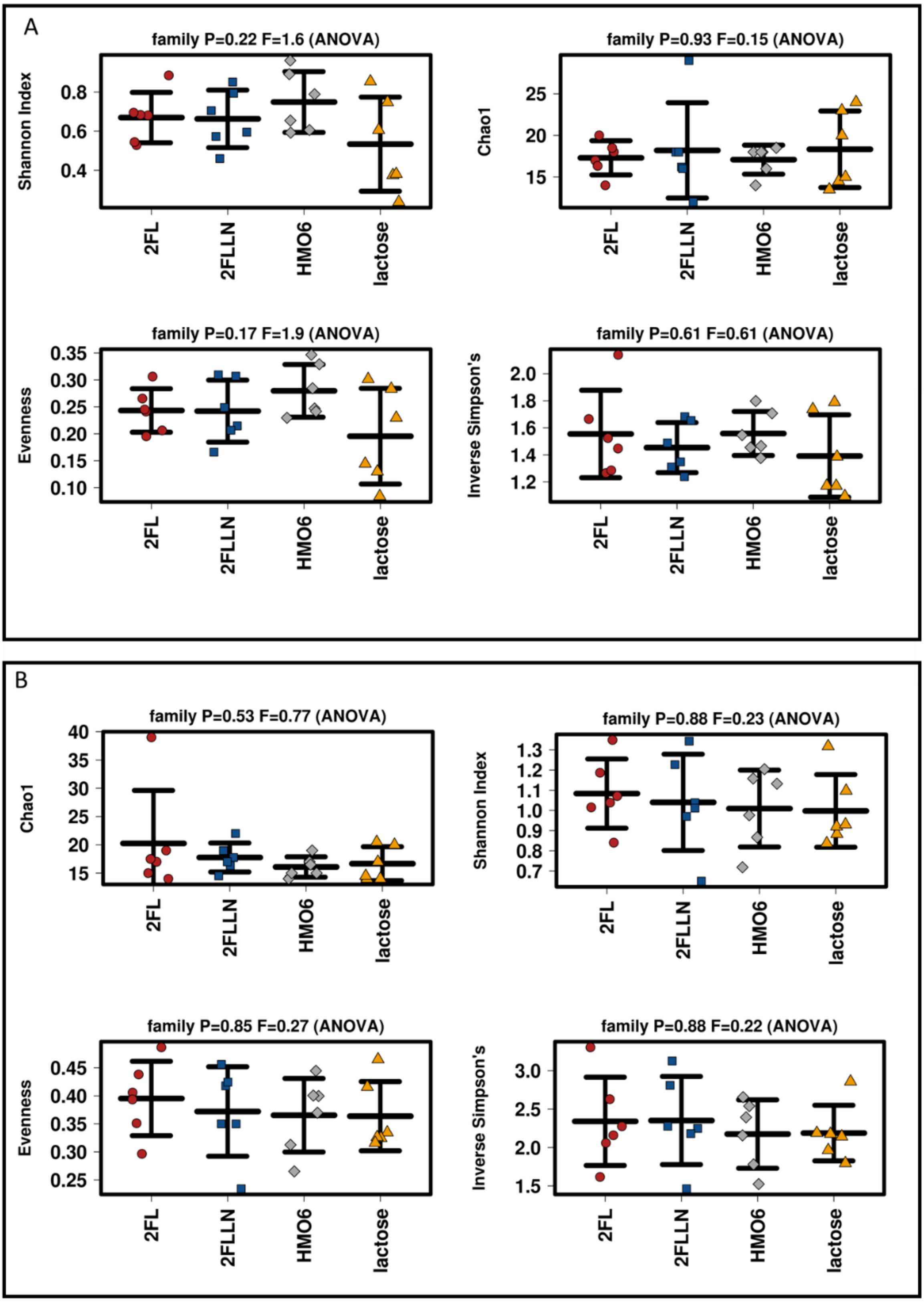

Figure S2: Effect of long-term exposure to lactose, 2'-FL, 2'-FL/LNnT and HMO6 on microbial community diversity markers at luminal (A) and mucosal (B) compartments of the distal colon at family level.

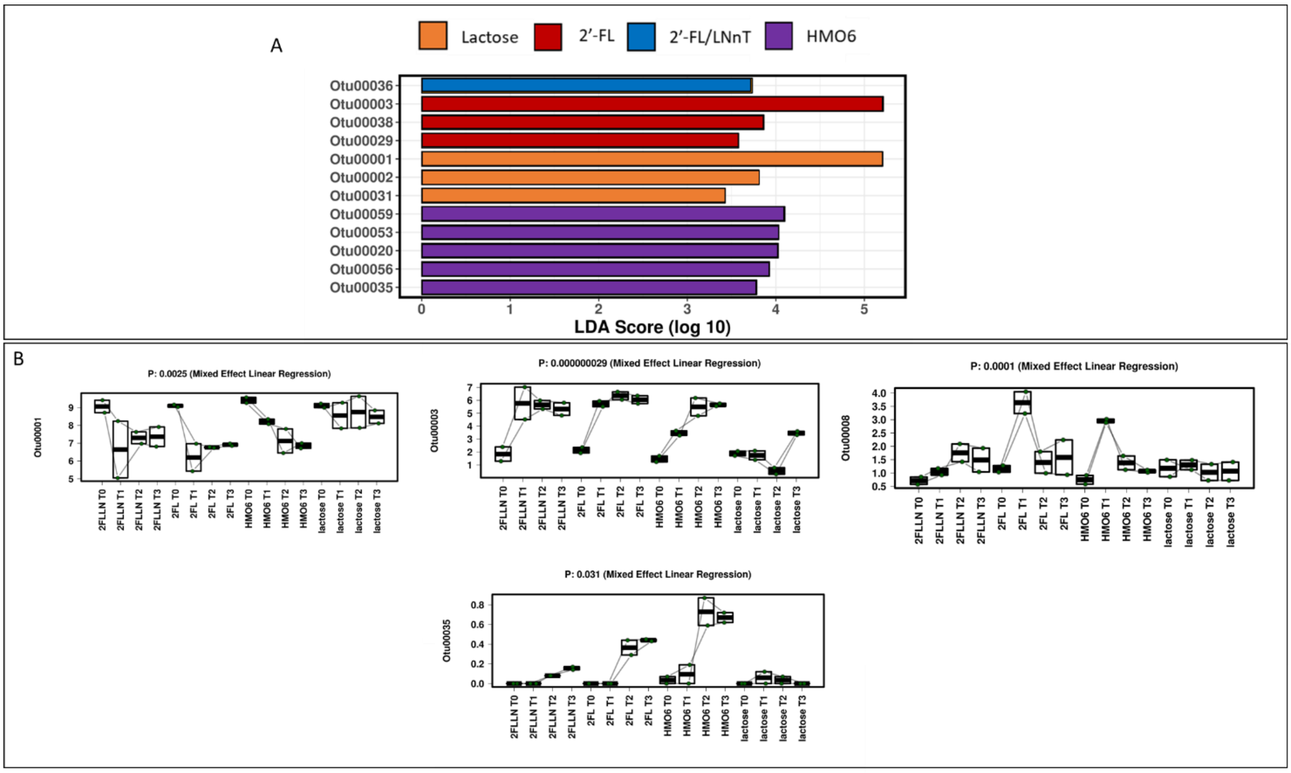

Figure S3: Modulation of microbiota by lactose, 2'-FL, 2'-FL/LNnT and HMO6 in the luminal compartment at OTU level. LEfSe analysis (A) and selected significant mixed effect regression plots (B) representing the effect of time and treatment on microbial community. Different sampling points are indicated by T0 (0 h), T1 (1 week of treatment), T2 (2 weeks of treatment), T3 (3 weeks of treatment). LEfSe analysis was performed using treatment time points (T1, T2, and T3). 2FLLN = 2'-fucosyllactose and lacto-N-neotetraose; 2FL = 2'-fucosyllactose; HMO6 = 2'-FL, LNnT, difucosyllactose, lacto-N-tetraose, 3'- and 6'-sialyllactose.

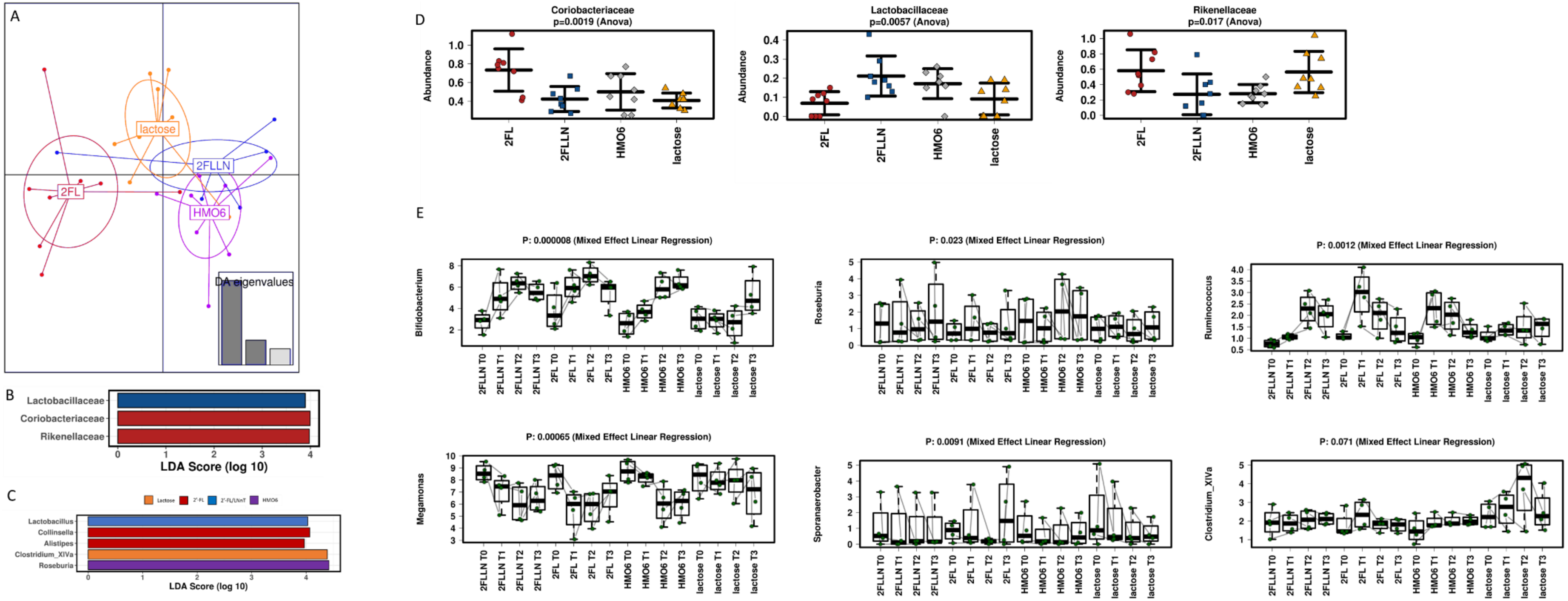

Figure S4: Effect of lactose, 2'-FL, 2'-FL/LNnT and HMO6 gut microbial community at family and genus level in the mucosal compartment of the long-term M-SHIME®. Discriminant Analysis of Principal Components (DAPC) plot (A); Linear discriminant analysis Effect Size (LEfSe) analysis at family level (B); LEfSe analysis at genus level (C); Strip chart plots of selected families with significant differences between treatments (D); Box plots of genus with significant differences between treatments in a mixed effect linear regression analysis including time and treatment as variables (E).

A

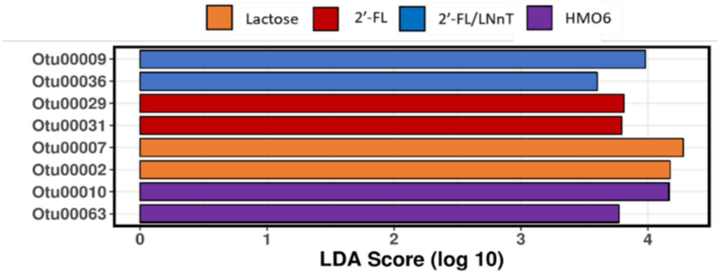

B

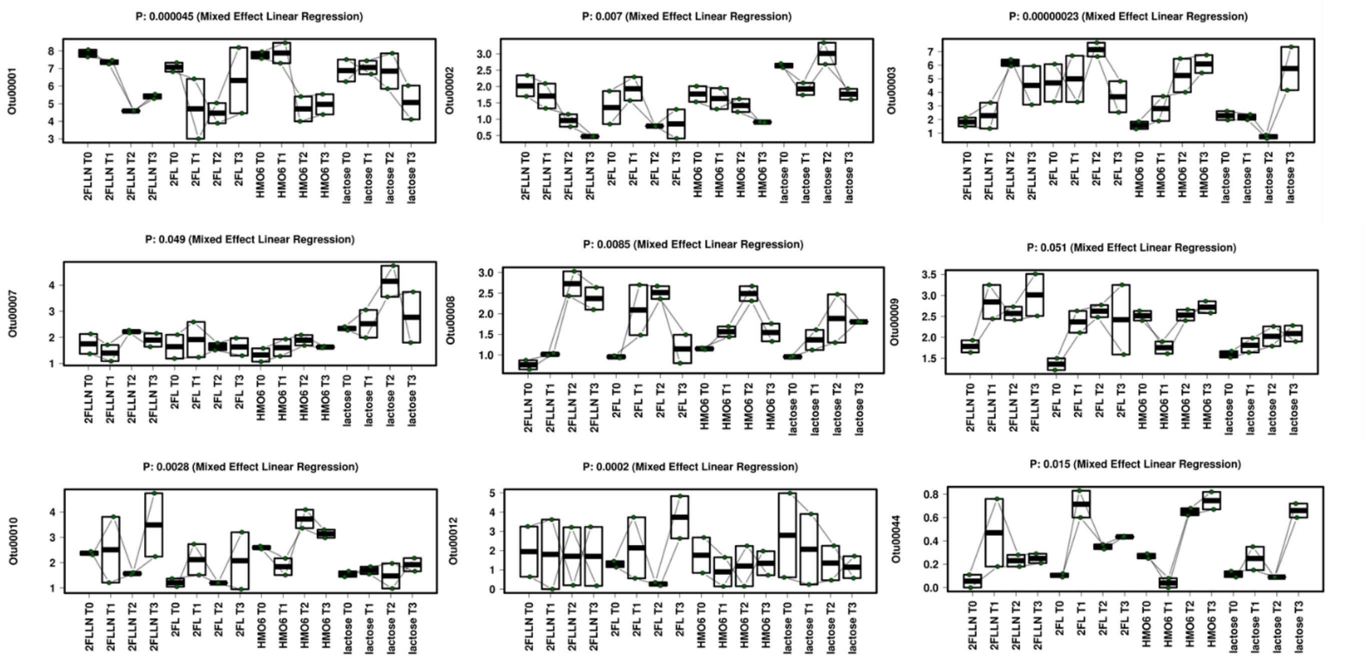

Figure S5: Effect of lactose, 2'-FL, 2'-FL/LNnT and HMO6 gut microbial community at OTU level in the mucosal compartment of the long-term M-SHIME®. Linear discriminant analysis Effect Size (LEfSe) analysis at OTU level (A); Selected significant features from Mixed effect regression analysis including time and treatment as variables (B).

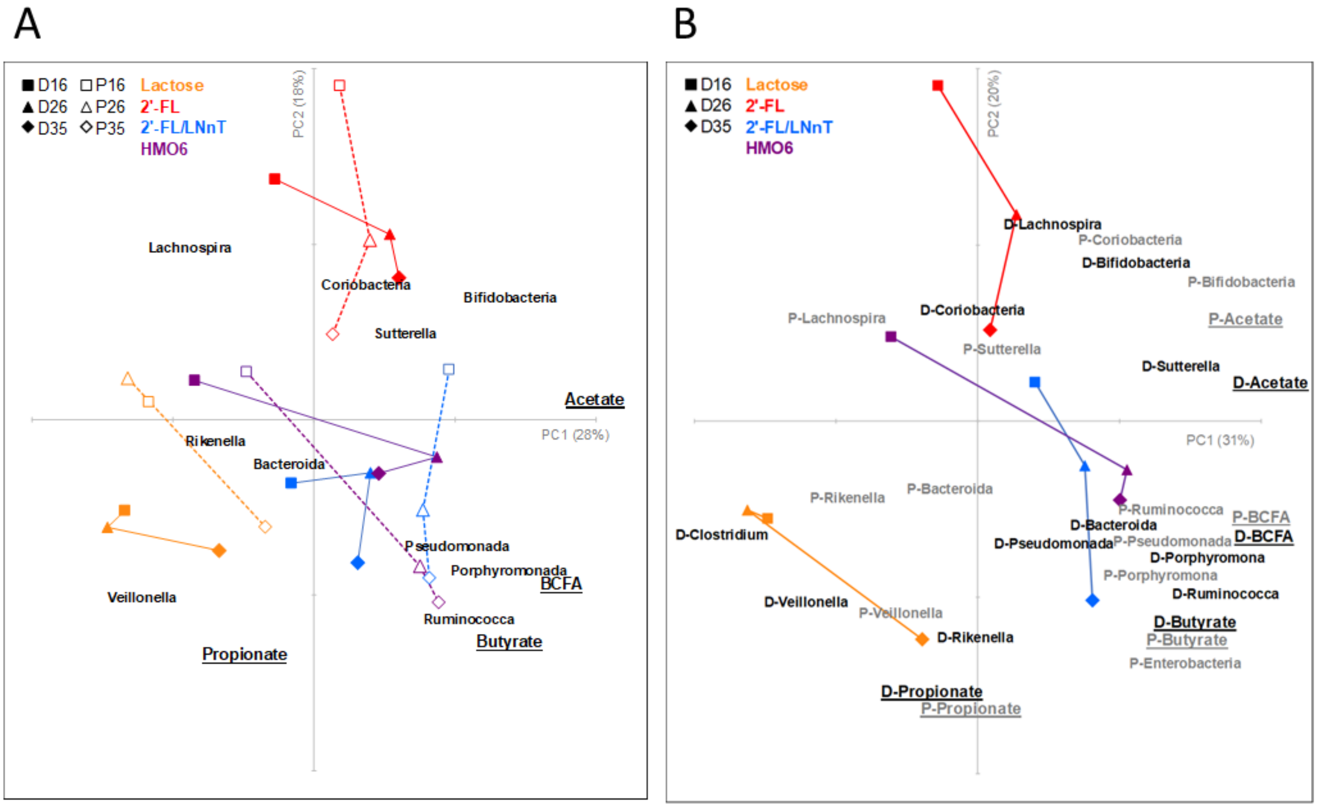

Figure S6: Principal component regression plots of short chain fatty acids and microbiota composition by 16S RNA seq considering proximal and distal colon compartment as different conditions (A) or analyzed together (B).

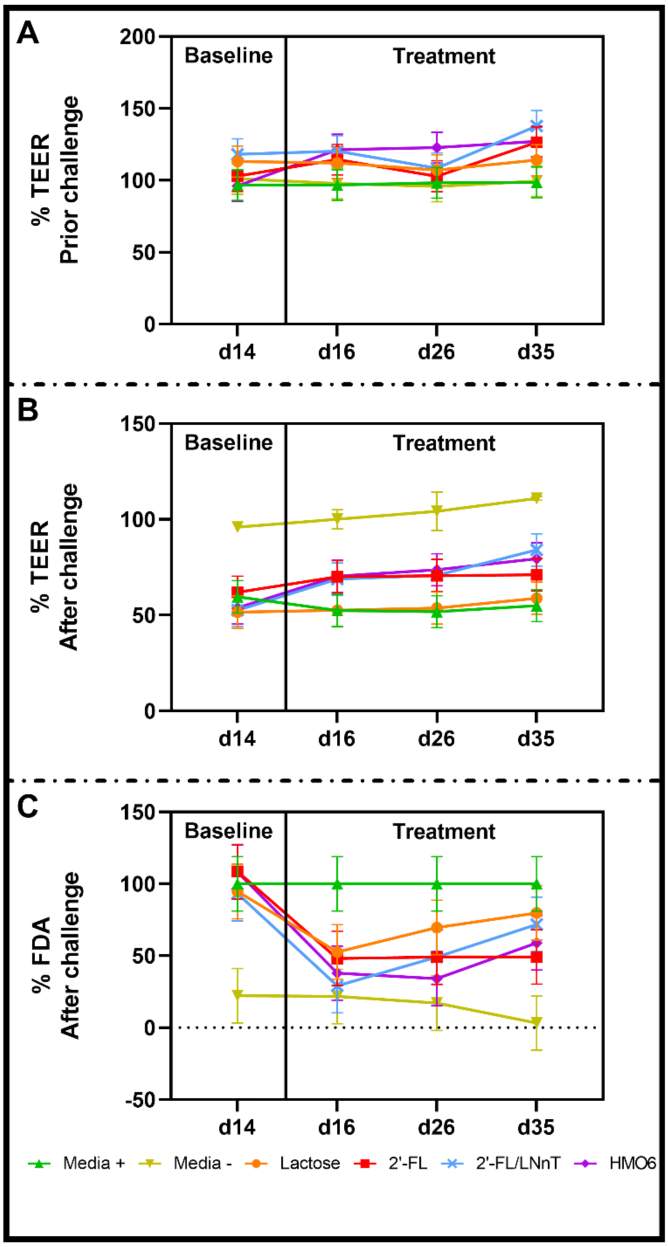

Figure S7: Time course effect of lactose, 2’-FL, 2’-FL/LNnT and HMO6 fermentation products on intestinal barrier function in vitro. Percentage of TEER values in intact (A) or IFNγ/TNFα-challenged (B) Caco-2/HT29-MTX monolayers exposed to baby M-SHIME® supernatants (36 h) from 2 (d16), 12 (d26) and 21 (d35) days of treatment. (C) Percentage of FD4 transport to the basolateral compartment in IFNγ/TNFα-challenged Caco-2/HT29-MTX monolayers exposed to baby M-SHIME® supernatants (36 h) from 2 (d16), 12 (d26) and 21 (d35) days of treatment.

Table S1: Effect of lactose, 2'-FL, 2'-FL/LNnT and HMO6 on bacterial activity markers and quantification of bifidobacteria by qPCR on five individual donors in short-term incubations in vitro. Data represent the value at start (t_0), end (t_{48}) and the delta change values (Δ48 h, single measure) of incubation in different reactors containing fecal samples from five different donors, except for the gas (T_0 = 0 kPa) and BCFA (T_0 = 0 mM) production.

		pH (upH)			Gas (kPa) (t_0=0)	Acetate (mM)			Propionate (mM)			Butyrate (mM)			BCFA (mM) (t_0=0)	Lactate (mM)			Ammonium (mg/L)			*Bifidobacteria* Log(copies/mL)
		t_0	t_{48}	Δ48 h	Δ48 h	t_0	t_{48}	Δ48 h	t_0	t_{48}	Δ48 h	t_0	t_{48}	Δ48 h	Δ48 h	t_0	t_{48}	Δ48 h	t_0	t_{48}	Δ48 h	48 h
Donor 1	Control	6.64	6.44	-0.20	57.7	0.01	4.18	4.17	0.00	2.15	2.14	0.00	0.00	0.00	1.62	0.00	1.62	0.00	0.01	6.33	6.32	10.56
	Lactose	6.67	4.75	-1.92	127.5	0.01	8.03	8.02	0.00	0.27	0.27	0.00	0.00	0.00	10.48	0.00	10.49	0.00	0.01	8.30	8.29	9.88
	2'-FL	6.63	6.45	-0.18	55.0	0.01	6.81	6.81	0.00	4.00	4.00	0.00	1.09	1.09	1.75	0.00	1.76	0.09	0.01	11.99	11.98	10.85
	2'-FL/LNnT	6.64	6.11	-0.53	93.4	0.01	13.80	13.80	0.00	4.99	4.99	0.00	0.52	0.52	1.95	0.00	1.96	0.00	0.01	19.32	19.31	11.48
	HMO6	6.64	6.23	-0.41	84.4	0.01	13.88	13.87	0.00	3.82	3.82	0.00	0.12	0.12	1.93	0.00	1.93	0.00	0.01	17.81	17.81	11.44
Donor 2	Control	6.65	6.41	-0.24	60.2	0.05	4.30	4.25	0.00	2.39	2.39	0.00	1.82	1.82	0.51	0.02	0.53	0.00	0.06	8.51	8.46	11.47
	Lactose	6.60	4.90	-1.66	158.9	0.05	20.38	20.33	0.00	0.24	0.23	0.00	0.00	0.00	9.39	0.02	9.41	0.00	0.06	20.62	20.56	12.33
	2'-FL	6.63	5.43	-1.20	79.8	0.05	24.35	24.30	0.00	10.80	10.80	0.00	2.87	2.86	1.71	0.02	1.72	0.00	0.06	38.02	37.96	10.58
	2'-FL/LNnT	6.63	6.02	-0.61	84.4	0.05	13.77	13.72	0.00	5.42	5.41	0.00	6.47	6.46	0.81	0.02	0.83	0.00	0.06	25.66	25.60	12.42
	HMO6	6.64	6.25	-0.39	72.0	0.05	9.62	9.56	0.00	4.61	4.61	0.00	3.85	3.85	0.95	0.02	0.97	0.00	0.06	18.08	18.02	12.22
Donor 3	Control	6.64	6.39	-0.25	46.6	0.04	6.50	6.46	0.00	0.53	0.53	0.00	0.00	0.00	0.78	0.03	0.81	0.00	0.04	7.03	6.99	10.90
	Lactose	6.60	5.52	-1.08	186.7	0.04	13.40	13.36	0.00	0.00	0.00	0.00	0.00	0.00	8.74	0.03	8.77	0.00	0.04	13.40	13.36	11.22
	2'-FL	6.64	4.94	-1.70	87.5	0.04	24.42	24.38	0.00	6.27	6.27	0.00	1.68	1.68	8.65	0.03	8.68	0.00	0.04	32.37	32.33	11.37
	2'-FL/LNnT	6.65	5.10	-1.55	116.1	0.04	23.75	23.71	0.00	7.82	7.82	0.00	0.58	0.57	8.90	0.03	8.93	0.05	0.04	32.20	32.16	11.59
	HMO6	6.63	5.12	-1.51	125.8	0.04	24.13	24.10	0.00	3.95	3.95	0.00	1.85	1.85	8.58	0.03	8.60	0.08	0.04	30.01	29.97	11.56
Donor 4	Control	6.61	6.40	-0.21	44.3	0.11	12.58	12.47	0.02	2.98	2.96	0.01	1.20	1.19	0.27	0.03	0.29	0.00	0.13	16.75	16.62	11.25
	Lactose	6.59	4.91	-1.68	98.1	0.11	32.54	32.44	0.02	3.54	3.52	0.01	0.14	0.13	9.23	0.03	9.23	0.00	0.13	36.22	36.09	11.69
	2'-FL	6.59	4.80	-1.79	97.8	0.11	42.39	42.28	0.02	3.62	3.60	0.01	0.35	0.34	5.99	0.03	6.01	0.00	0.13	46.35	46.22	12.46
	2'-FL/LNnT	6.60	4.78	-1.82	91.5	0.11	41.74	41.63	0.02	3.32	3.30	0.01	0.77	0.76	7.53	0.03	7.56	0.00	0.13	45.82	45.69	12.31
	HMO6	6.61	5.07	-1.54	125.4	0.11	43.96	43.86	0.02	3.61	3.59	0.01	1.30	1.29	5.77	0.03	5.79	0.00	0.13	48.88	48.74	12.56
Donor 5	Control	6.63	6.49	-0.14	62.0	0.03	7.25	7.22	0.01	3.92	3.91	0.01	1.84	1.84	0.44	0.02	0.46	1.37	0.05	14.38	14.33	10.79
	Lactose	6.65	5.06	-1.59	106.9	0.03	20.57	20.54	0.01	2.93	2.91	0.01	0.00	-0.01	9.23	0.02	9.25	0.00	0.05	23.50	23.45	11.93
	2'-FL	6.65	5.53	-1.12	115.1	0.03	22.42	22.39	0.01	17.09	17.07	0.01	4.57	4.56	0.77	0.02	0.79	1.08	0.05	45.16	45.11	11.87
	2'-FL/LNnT	6.67	5.52	-1.15	125.1	0.03	26.82	26.79	0.01	10.96	10.95	0.01	2.57	2.56	0.53	0.02	0.55	1.21	0.05	41.56	41.51	12.32
	HMO6	6.65	5.56	-1.09	120.7	0.03	26.39	26.36	0.01	12.57	12.56	0.01	30.5	3.04	0.46	0.02	0.47	1.31	0.05	43.32	43.27	12.31

Table S2: Proportional microbial composition at the family level (%) as determined via 16S-targeted Illumina sequencing in the lumen of the proximal and distal colon compartments of the M-SHIME® before (d14) and after (d16, d26, and d35) treatment with lactose, 2’-FL, 2’-FL/LNnT or HMO6. The intensity of the shading indicates the absolute abundance, normalized for each of the different families within each row.

	Phylum	Family	Lactose				2'-FL				2'-FL/LNnT				HMO6			
			d14	d16	d26	d35	d14	d16	d26	d35	d14	d16	d26	d35	d14	d16	d26	d35
**Proximal colon (PC)**	**Actinobacteria**	***Bifidobacteriaceae***	5.3%	8.2%	4.4%	16.8%	6.5%	38.0%	38.2%	34.9%	9.0%	58.4%	37.1%	35.3%	3.5%	15.2%	25.1%	33.2%
		Coriobacteriaceae	0.3%	0.1%	0.1%	0.1%	0.2%	0.2%	0.6%	0.3%	0.2%	0.1%	0.0%	0.0%	0.2%	0.1%	0.3%	0.4%
	**Bacteroidetes**	***Bacteroidaceae***	1.4%	6.7%	0.5%	1.1%	2.1%	1.6%	1.3%	0.8%	2.7%	1.0%	1.5%	1.5%	1.7%	1.3%	1.4%	3.0%
		Porphyromonadaceae	0.1%	0.3%	0.2%	0.3%	0.3%	0.2%	0.2%	0.2%	0.3%	0.5%	0.3%	0.8%	0.2%	0.3%	0.8%	0.2%
		Rikenellaceae	0.1%	0.2%	0.7%	0.3%	0.1%	0.1%	0.2%	0.5%	0.0%	0.0%	0.0%	0.1%	0.0%	0.0%	0.0%	0.1%
	**Firmicutes**	***Clostridium* cluster I**	0.0%	0.0%	0.0%	0.0%	0.0%	0.0%	0.0%	0.0%	0.0%	0.0%	0.0%	0.0%	0.0%	0.0%	0.0%	0.0%
		***Clostridium* cluster XI**	0.0%	0.1%	0.0%	0.0%	0.0%	0.0%	0.0%	0.0%	0.0%	0.0%	0.0%	0.0%	0.0%	0.0%	0.0%	0.0%
		Lachnospiraceae	4.1%	14.4%	26.8%	9.8%	4.0%	26.1%	7.5%	10.7%	5.1%	7.9%	8.0%	10.4%	3.1%	13.4%	5.5%	8.3%
		Lactobacillaceae	0.0%	0.0%	0.0%	0.0%	0.0%	0.0%	0.0%	0.0%	0.0%	0.0%	0.0%	0.0%	0.0%	0.0%	0.0%	0.0%
		Ruminococcaceae	0.4%	1.3%	0.2%	0.5%	0.8%	0.2%	0.1%	0.2%	0.7%	0.5%	1.2%	0.5%	1.5%	0.4%	2.3%	4.9%
		Veillonellaceae	86.4%	66.0%	64.7%	68.6%	83.1%	32.0%	49.8%	49.8%	79.5%	30.1%	49.9%	48.3%	86.9%	67.0%	62.4%	47.7%
	**Proteobacteria**	***Enterobacteriaceae***	0.0%	0.0%	0.0%	0.1%	0.0%	0.0%	0.0%	0.0%	0.0%	0.0%	0.0%	0.1%	0.0%	0.0%	0.1%	0.1%
		Pseudomonadaceae	0.1%	0.1%	0.2%	0.3%	0.1%	0.1%	0.4%	0.1%	0.1%	0.1%	0.8%	0.6%	0.1%	0.0%	0.2%	0.2%
		Sutterellaceae	0.1%	0.1%	0.1%	0.1%	0.2%	0.1%	0.2%	0.1%	0.1%	0.0%	0.0%	0.1%	0.1%	0.0%	0.1%	0.0%
**Distal colon (DC)**	**Actinobacteria**	***Bifidobacteriaceae***	3.6%	2.8%	0.6%	12.4%	4.3%	31.6%	45.5%	41.8%	2.3%	21.5%	29.4%	24.1%	1.8%	11.6%	41.5%	35.3%
		Coriobacteriaceae	0.3%	0.1%	0.0%	0.1%	0.3%	0.1%	0.7%	1.4%	0.2%	0.1%	0.1%	0.1%	0.1%	0.0%	0.3%	0.2%
	**Bacteroidetes**	***Bacteroidaceae***	1.4%	0.9%	0.2%	0.5%	1.5%	0.6%	0.3%	0.4%	1.0%	0.7%	0.6%	1.0%	1.0%	0.9%	1.2%	2.2%
		Porphyromonadaceae	0.2%	0.1%	0.1%	0.3%	0.3%	0.1%	0.2%	0.2%	0.2%	0.2%	0.2%	0.7%	0.1%	0.3%	0.8%	0.2%
		Rikenellaceae	0.1%	0.1%	0.1%	0.4%	0.0%	0.0%	0.1%	0.2%	0.0%	0.0%	0.1%	0.2%	0.0%	0.0%	0.0%	0.1%
	**Firmicutes**	***Clostridium* cluster I**	0.0%	0.0%	0.0%	0.0%	0.0%	0.0%	0.0%	0.0%	0.0%	0.0%	0.0%	0.0%	0.0%	0.0%	0.0%	0.0%
		***Clostridium* cluster XI**	1.2%	0.3%	0.1%	0.1%	0.3%	0.0%	0.0%	0.1%	0.1%	0.0%	0.0%	0.0%	0.0%	0.0%	0.0%	0.0%
		Lachnospiraceae	6.5%	4.4%	2.9%	3.8%	4.8%	16.5%	3.5%	3.8%	2.5%	4.9%	5.9%	5.4%	1.8%	14.0%	8.5%	7.2%
		Lactobacillaceae	0.0%	0.0%	0.0%	0.0%	0.0%	0.0%	0.0%	0.0%	0.0%	0.0%	0.0%	0.0%	0.0%	0.0%	0.0%	0.0%
		Ruminococcaceae	1.1%	1.0%	0.5%	0.7%	0.8%	0.2%	0.2%	0.2%	1.0%	1.6%	2.2%	1.5%	0.4%	0.8%	2.7%	2.2%
		Veillonellaceae	82.8%	88.3%	93.1%	79.4%	85.2%	49.6%	47.5%	50.1%	90.3%	69.3%	59.2%	64.6%	92.2%	69.7%	43.2%	50.5%
	**Proteobacteria**	***Enterobacteriaceae***	0.0%	0.0%	0.0%	0.0%	0.0%	0.0%	0.0%	0.0%	0.0%	0.0%	0.0%	0.0%	0.0%	0.0%	0.0%	0.0%
		Pseudomonadaceae	0.1%	0.1%	0.3%	0.2%	0.0%	0.0%	0.3%	0.1%	0.1%	0.2%	0.3%	0.2%	0.0%	0.0%	0.1%	0.1%
		Sutterellaceae	0.3%	0.1%	0.0%	0.1%	0.2%	0.1%	0.2%	0.1%	0.2%	0.1%	0.2%	0.1%	0.1%	0.0%	0.1%	0.1%

Table S3: Proportional microbial composition at the OTU level (%) as determined via 16S-targeted Illumina sequencing in the lumen of the proximal colon compartment of the M-SHIME® before (d14) and after (d16, d26, and d35) treatment with lactose, 2’-FL, 2’-FL/LNnT or HMO6. The intensity of the shading indicates the absolute abundance, normalized for each OTUs within each row.

				Lumen - PC															
Phylum	Family	OTU number	Related species	Lactose				2'-FL				2'-FL/LNnT				HMO6			
				d14	d16	d26	d35	d14	d16	d26	d35	d14	d16	d26	d35	d14	d16	d26	d35
Actinobacteria	*Bifidobacteriaceae*	2	*Bifidobacterium longum*	0.7%	2.9%	3.0%	2.7%	0.6%	1.7%	0.1%	0.1%	1.9%	5.6%	0.2%	0.1%	0.4%	1.4%	0.8%	0.3%
		3	*Bifidobacterium adolescentis*	4.3%	4.4%	0.6%	13.0%	5.6%	35.3%	36.6%	33.0%	5.7%	49.3%	35.8%	33.7%	2.8%	13.2%	23.1%	30.9%
		9	*Bifidobacterium bifidum*	0.4%	0.9%	0.8%	0.9%	0.3%	0.9%	1.3%	1.6%	1.3%	2.9%	1.1%	1.5%	0.2%	0.5%	0.8%	1.4%
		35	*Bifidobacterium dentium*	0.0%	0.0%	0.0%	0.0%	0.0%	0.0%	0.2%	0.2%	0.0%	0.0%	0.0%	0.0%	0.0%	0.0%	0.3%	0.4%
		44	*Bifidobacterium bifidum*	0.0%	0.0%	0.0%	0.2%	0.0%	0.1%	0.0%	0.0%	0.0%	0.6%	0.0%	0.0%	0.0%	0.1%	0.0%	0.1%
	Coriobacteriaceae	29	*Collinsella aerofaciens*	0.3%	0.1%	0.1%	0.1%	0.2%	0.2%	0.6%	0.3%	0.2%	0.1%	0.0%	0.0%	0.2%	0.1%	0.3%	0.4%
Bacteroidetes	*Bacteroidaceae*	22	*Bacteroides fragilis*	0.6%	3.3%	0.4%	0.9%	0.5%	0.5%	1.1%	0.6%	0.6%	0.3%	0.8%	1.0%	0.3%	0.8%	1.1%	2.5%
		21	*Bacteroides xylanisolvens*	0.7%	2.7%	0.1%	0.2%	1.1%	0.8%	0.2%	0.1%	1.6%	0.5%	0.5%	0.2%	1.0%	0.3%	0.3%	0.5%
		45	*Bacteroides thetaiotaomicron*	0.1%	0.4%	0.0%	0.0%	0.2%	0.1%	0.0%	0.0%	0.2%	0.1%	0.1%	0.1%	0.1%	0.1%	0.1%	0.0%
		50	*Bacteroides caccae*	0.1%	0.2%	0.0%	0.0%	0.2%	0.2%	0.0%	0.0%	0.2%	0.0%	0.1%	0.1%	0.1%	0.0%	0.0%	0.0%
		48	*Bacteroides uniformis*	0.0%	0.1%	0.0%	0.0%	0.1%	0.0%	0.0%	0.0%	0.1%	0.0%	0.0%	0.1%	0.1%	0.0%	0.0%	0.0%
	Porphyromonadaceae	26	*Parabacteroides distasonis*	0.1%	0.3%	0.2%	0.3%	0.3%	0.2%	0.2%	0.2%	0.3%	0.5%	0.3%	0.8%	0.2%	0.3%	0.8%	0.2%
	Rikenellaceae	31	*Allistipes finegoldii*	0.1%	0.2%	0.7%	0.3%	0.1%	0.1%	0.2%	0.5%	0.0%	0.0%	0.0%	0.1%	0.0%	0.0%	0.0%	0.1%
Firmicutes	Clostridium cluster I	46	*Clostridium cadaveris*	0.0%	0.0%	0.0%	0.0%	0.0%	0.0%	0.0%	0.0%	0.0%	0.0%	0.0%	0.0%	0.0%	0.0%	0.0%	0.0%
	Clostridium cluster XI	12	*Sporanaerobacter acetigenes*	0.0%	0.1%	0.0%	0.0%	0.0%	0.0%	0.0%	0.0%	0.0%	0.0%	0.0%	0.0%	0.0%	0.0%	0.0%	0.0%
	Lachnospiraceae	7	*Clostridium clostridioforme*	2.4%	9.1%	23.3%	5.4%	1.3%	7.1%	3.0%	3.7%	3.2%	5.6%	2.4%	5.1%	0.9%	2.5%	2.8%	4.7%
		10	*Roseburia inulinivorans*	0.0%	0.4%	0.1%	0.2%	0.1%	0.1%	0.1%	0.1%	0.0%	0.1%	0.1%	0.2%	0.0%	0.1%	0.1%	0.1%
		8	*Ruminococcus torques/faecis*	0.7%	2.2%	1.8%	2.0%	1.1%	16.4%	3.2%	5.0%	0.3%	0.9%	4.4%	3.7%	0.8%	9.1%	1.3%	1.0%
		42	*Eubacterium rectale*	0.0%	0.0%	0.0%	0.0%	0.0%	0.0%	0.0%	0.0%	0.0%	0.0%	0.0%	0.0%	0.0%	0.0%	0.0%	0.0%
		18	Butyrate-producing bacterium SS3/4	0.3%	0.9%	0.5%	0.7%	0.3%	0.1%	0.4%	0.6%	0.3%	0.2%	0.2%	0.3%	0.1%	0.2%	0.3%	0.5%
		27	*Faecalicatena fissicatena*	0.0%	0.0%	0.0%	0.0%	0.0%	0.0%	0.0%	0.0%	0.0%	0.0%	0.0%	0.0%	0.0%	0.0%	0.0%	0.0%
		19	Butyrate-producing bacterium SR1/5	0.1%	0.3%	0.0%	0.2%	0.2%	1.0%	0.0%	0.1%	0.1%	0.5%	0.1%	0.0%	0.1%	0.7%	0.1%	0.1%
		24	*Clostridium scindens*	0.2%	0.2%	0.2%	0.2%	0.3%	0.1%	0.0%	0.1%	0.4%	0.2%	0.0%	0.0%	0.1%	0.0%	0.0%	0.0%
		28	*Eubacterium ramulus*	0.0%	0.0%	0.0%	0.3%	0.0%	0.0%	0.2%	0.3%	0.0%	0.0%	0.3%	0.3%	0.0%	0.0%	0.4%	0.7%
		23	*Lachnoclostridium sp.*	0.3%	0.6%	0.5%	0.2%	0.4%	0.9%	0.4%	0.3%	0.3%	0.1%	0.3%	0.3%	0.5%	0.4%	0.3%	0.6%
		40	*Blautia wexlerae*	0.1%	0.1%	0.0%	0.0%	0.0%	0.0%	0.0%	0.0%	0.2%	0.1%	0.0%	0.0%	0.2%	0.0%	0.0%	0.0%
		41	*Roseburia hominis*	0.0%	0.0%	0.0%	0.0%	0.0%	0.0%	0.0%	0.0%	0.0%	0.0%	0.0%	0.0%	0.0%	0.0%	0.0%	0.0%
		33	*Blautia producta*	0.0%	0.0%	0.0%	0.0%	0.0%	0.0%	0.0%	0.0%	0.0%	0.0%	0.0%	0.0%	0.0%	0.0%	0.0%	0.0%
		32	*Anaerobutyricum hallii*	0.0%	0.2%	0.2%	0.6%	0.0%	0.1%	0.1%	0.5%	0.0%	0.1%	0.2%	0.4%	0.1%	0.2%	0.2%	0.6%
		39	*Coprococcus comes*	0.1%	0.4%	0.1%	0.1%	0.3%	0.2%	0.0%	0.0%	0.1%	0.1%	0.0%	0.0%	0.1%	0.0%	0.0%	0.0%
	Lactobacillaceae	15	*Lactobacillus paracasei*	0.0%	0.0%	0.0%	0.0%	0.0%	0.0%	0.0%	0.0%	0.0%	0.0%	0.0%	0.0%	0.0%	0.0%	0.0%	0.0%
	Ruminococcaceae	20	*Faecalibacterium prausnitzii*	0.3%	1.2%	0.2%	0.5%	0.6%	0.1%	0.1%	0.1%	0.5%	0.5%	0.6%	0.3%	1.3%	0.4%	2.2%	4.7%
		36	*Clostridium* sp.	0.0%	0.0%	0.0%	0.0%	0.1%	0.0%	0.0%	0.0%	0.1%	0.0%	0.6%	0.2%	0.0%	0.0%	0.1%	0.1%
		34	*Oscilibacter massiliensis*	0.0%	0.0%	0.0%	0.0%	0.0%	0.0%	0.0%	0.0%	0.0%	0.0%	0.0%	0.0%	0.0%	0.0%	0.0%	0.0%
		47	*Ruthenibacterium lactatiformans*	0.0%	0.1%	0.0%	0.0%	0.1%	0.0%	0.0%	0.0%	0.1%	0.1%	0.0%	0.0%	0.1%	0.0%	0.0%	0.0%
		54	*Flavonifractor plautii*	0.0%	0.0%	0.0%	0.0%	0.0%	0.0%	0.0%	0.0%	0.0%	0.0%	0.0%	0.0%	0.0%	0.0%	0.0%	0.0%
	Veillonellaceae	1	*Megamonas funiformis*	85.1%	61.3%	61.8%	65.8%	81.5%	29.5%	45.5%	46.9%	75.9%	25.5%	48.6%	46.3%	85.5%	65.1%	60.8%	45.0%
		13	*Veillonella ratti*	1.3%	4.5%	2.8%	2.7%	1.5%	2.4%	4.3%	2.9%	3.5%	4.5%	1.3%	1.9%	1.3%	1.9%	1.6%	2.8%
		37	*Negativicoccus massiliensis*	0.0%	0.1%	0.0%	0.0%	0.1%	0.0%	0.0%	0.0%	0.1%	0.1%	0.0%	0.0%	0.1%	0.0%	0.0%	0.0%
Proteobacteria	*Enterobacteriaceae*	6	*Klebsiella oxytoca*	0.0%	0.0%	0.0%	0.1%	0.0%	0.0%	0.0%	0.0%	0.0%	0.0%	0.0%	0.1%	0.0%	0.0%	0.1%	0.1%
	Pseudomonadaceae	43	*Pseudomonas aeruginosa*	0.1%	0.1%	0.2%	0.3%	0.1%	0.1%	0.4%	0.1%	0.1%	0.1%	0.8%	0.6%	0.1%	0.0%	0.2%	0.2%
	Sutterellaceae	38	*Sutterella* sp.	0.1%	0.1%	0.1%	0.1%	0.2%	0.1%	0.2%	0.1%	0.1%	0.0%	0.0%	0.1%	0.1%	0.0%	0.1%	0.0%

Table S4: Proportional microbial composition at the OTU level (%) as determined via 16S-targeted Illumina sequencing in the lumen of the distal colon compartment of the M-SHIME® before (d14) and after (d16, d26, and d35) treatment with lactose, 2’-FL, 2’-FL/LNnT or HMO6. The intensity of the shading indicates the absolute abundance, normalized for each OTUs within each row.

				Lumen - DC															
Phylum	Family	OTU number	Related species	Lactose				2'-FL				2'-FL/LNnT				HMO6			
				d14	d16	d26	d35	d14	d16	d26	d35	d14	d16	d26	d35	d14	d16	d26	d35
Actinobacteria	*Bifidobacteriaceae*	2	*Bifidobacterium longum*	0.4%	0.5%	0.5%	0.9%	0.3%	0.8%	0.1%	0.1%	0.3%	0.3%	0.1%	0.0%	0.2%	0.4%	1.1%	0.3%
		3	*Bifidobacterium adolescentis*	2.9%	1.9%	0.1%	11.0%	3.7%	30.4%	44.2%	40.3%	1.7%	20.5%	28.5%	23.3%	1.5%	10.8%	38.2%	33.1%
		9	*Bifidobacterium bifidum*	0.2%	0.3%	0.1%	0.4%	0.3%	0.4%	1.1%	1.3%	0.4%	0.6%	0.9%	0.7%	0.1%	0.4%	1.2%	1.3%
		35	*Bifidobacterium dentium*	0.0%	0.0%	0.0%	0.0%	0.0%	0.0%	0.1%	0.2%	0.0%	0.0%	0.0%	0.0%	0.0%	0.0%	0.8%	0.5%
		44	*Bifidobacterium bifidum*	0.0%	0.0%	0.0%	0.0%	0.1%	0.0%	0.0%	0.0%	0.0%	0.1%	0.0%	0.0%	0.0%	0.0%	0.3%	0.1%
	Coriobacteriaceae	29	*Collinsella aerofaciens*	0.3%	0.1%	0.0%	0.1%	0.3%	0.1%	0.7%	1.4%	0.2%	0.1%	0.1%	0.1%	0.1%	0.0%	0.3%	0.2%
Bacteroidetes	*Bacteroidaceae*	22	*Bacteroides fragilis*	0.4%	0.4%	0.1%	0.3%	0.3%	0.2%	0.2%	0.2%	0.2%	0.3%	0.3%	0.8%	0.2%	0.5%	1.0%	1.9%
		21	*Bacteroides xylanisolvens*	0.8%	0.4%	0.0%	0.1%	0.8%	0.3%	0.1%	0.2%	0.6%	0.4%	0.3%	0.1%	0.6%	0.3%	0.2%	0.2%
		45	*Bacteroides thetaiotaomicron*	0.1%	0.1%	0.0%	0.0%	0.2%	0.1%	0.0%	0.0%	0.2%	0.0%	0.0%	0.1%	0.1%	0.1%	0.0%	0.0%
		50	*Bacteroides caccae*	0.0%	0.0%	0.0%	0.0%	0.1%	0.0%	0.0%	0.0%	0.0%	0.0%	0.0%	0.0%	0.0%	0.0%	0.0%	0.0%
		48	*Bacteroides uniformis*	0.1%	0.1%	0.0%	0.0%	0.1%	0.0%	0.0%	0.0%	0.1%	0.0%	0.0%	0.1%	0.0%	0.0%	0.0%	0.1%
	Porphyromonadaceae	26	*Parabacteroides distasonis*	0.2%	0.1%	0.1%	0.3%	0.3%	0.1%	0.2%	0.2%	0.2%	0.2%	0.2%	0.7%	0.1%	0.3%	0.8%	0.2%
	Rikenellaceae	31	*Allistipes finegoldii*	0.1%	0.1%	0.1%	0.4%	0.0%	0.0%	0.1%	0.2%	0.0%	0.0%	0.1%	0.2%	0.0%	0.0%	0.0%	0.1%
Firmicutes	Clostridium cluster I	46	*Clostridium cadaveris*	0.0%	0.0%	0.0%	0.0%	0.0%	0.0%	0.0%	0.0%	0.0%	0.0%	0.0%	0.0%	0.0%	0.0%	0.0%	0.0%
	Clostridium cluster XI	12	*Sporanaerobacter acetigenes*	1.2%	0.3%	0.1%	0.1%	0.3%	0.0%	0.0%	0.1%	0.1%	0.0%	0.0%	0.0%	0.0%	0.0%	0.0%	0.0%
	Lachnospiraceae	7	*Clostridium clostridioforme*	1.8%	1.5%	1.8%	1.9%	0.9%	1.8%	1.4%	1.3%	0.7%	1.5%	2.0%	2.8%	0.3%	2.2%	2.6%	2.5%
		10	*Roseburia inulinivorans*	0.1%	0.2%	0.0%	0.1%	0.1%	0.1%	0.0%	0.2%	0.0%	0.0%	0.1%	0.1%	0.0%	0.0%	0.2%	0.1%
		8	*Ruminococcus torques/faecis*	2.2%	1.3%	0.5%	0.5%	1.6%	10.4%	1.0%	0.9%	0.7%	1.4%	2.0%	1.1%	0.3%	8.4%	2.7%	1.2%
		42	*Eubacterium rectale*	0.0%	0.0%	0.0%	0.0%	0.0%	0.0%	0.0%	0.0%	0.0%	0.0%	0.0%	0.0%	0.0%	0.0%	0.0%	0.0%
		18	Butyrate-producing bacterium SS3/4	0.5%	0.4%	0.2%	0.3%	0.3%	0.3%	0.4%	0.2%	0.3%	0.4%	0.5%	0.3%	0.1%	0.3%	0.5%	0.5%
		27	*Faecalicatena fissicatena*	0.0%	0.0%	0.0%	0.0%	0.0%	0.0%	0.0%	0.0%	0.0%	0.0%	0.0%	0.0%	0.0%	0.0%	0.0%	0.0%
		19	Butyrate-producing bacterium SR1/5	0.4%	0.3%	0.0%	0.1%	0.3%	2.4%	0.0%	0.1%	0.2%	0.7%	0.3%	0.1%	0.1%	1.6%	0.3%	0.1%
		24	*Clostridium scindens*	0.2%	0.1%	0.1%	0.1%	0.3%	0.1%	0.0%	0.1%	0.1%	0.1%	0.1%	0.1%	0.0%	0.0%	0.2%	0.1%
		28	*Eubacterium ramulus*	0.0%	0.0%	0.0%	0.3%	0.0%	0.0%	0.1%	0.3%	0.0%	0.1%	0.3%	0.4%	0.0%	0.0%	1.5%	1.5%
		23	*Lachnoclostridium sp.*	0.7%	0.4%	0.1%	0.2%	0.5%	0.4%	0.3%	0.3%	0.3%	0.3%	0.3%	0.3%	0.2%	0.6%	0.3%	0.5%
		40	*Blautia wexlerae*	0.2%	0.0%	0.0%	0.0%	0.3%	0.3%	0.0%	0.0%	0.2%	0.3%	0.0%	0.0%	0.5%	0.5%	0.0%	0.0%
		41	*Roseburia hominis*	0.0%	0.0%	0.0%	0.0%	0.0%	0.0%	0.0%	0.0%	0.0%	0.0%	0.0%	0.0%	0.0%	0.0%	0.0%	0.0%
		33	*Blautia producta*	0.1%	0.0%	0.0%	0.1%	0.2%	0.3%	0.0%	0.0%	0.0%	0.0%	0.0%	0.1%	0.0%	0.1%	0.0%	0.1%
		32	*Anaerobutyricum hallii*	0.0%	0.1%	0.1%	0.2%	0.0%	0.1%	0.2%	0.2%	0.0%	0.1%	0.4%	0.2%	0.0%	0.2%	0.2%	0.5%
		39	*Coprococcus comes*	0.3%	0.1%	0.0%	0.0%	0.4%	0.2%	0.0%	0.0%	0.1%	0.0%	0.0%	0.0%	0.1%	0.0%	0.0%	0.0%
	Lactobacillaceae	15	*Lactobacillus paracasei*	0.0%	0.0%	0.0%	0.0%	0.0%	0.0%	0.0%	0.0%	0.0%	0.0%	0.0%	0.0%	0.0%	0.0%	0.0%	0.0%
	Ruminococcaceae	20	*Faecalibacterium prausnitzii*	0.8%	0.9%	0.5%	0.6%	0.6%	0.1%	0.1%	0.2%	0.8%	1.1%	0.9%	0.4%	0.4%	0.7%	2.6%	2.1%
		36	*Clostridium* sp.	0.0%	0.0%	0.0%	0.0%	0.0%	0.0%	0.0%	0.0%	0.0%	0.5%	1.3%	1.1%	0.0%	0.0%	0.0%	0.1%
		34	*Oscilibacter massiliensis*	0.1%	0.0%	0.0%	0.0%	0.0%	0.0%	0.0%	0.0%	0.0%	0.0%	0.0%	0.0%	0.0%	0.1%	0.0%	0.0%
		47	*Ruthenibacterium lactatiformans*	0.1%	0.1%	0.0%	0.0%	0.2%	0.1%	0.0%	0.0%	0.1%	0.0%	0.0%	0.0%	0.0%	0.0%	0.0%	0.0%
		54	*Flavonifractor plautii*	0.0%	0.0%	0.0%	0.0%	0.0%	0.0%	0.0%	0.0%	0.0%	0.0%	0.0%	0.0%	0.0%	0.0%	0.0%	0.0%
	Veillonellaceae	1	*Megamonas funiformis*	80.8%	85.8%	92.7%	78.1%	83.9%	48.5%	46.1%	48.7%	88.6%	67.9%	58.2%	62.6%	91.5%	69.7%	41.6%	49.0%
		13	*Veillonella ratti*	1.9%	2.4%	0.4%	1.3%	1.2%	1.1%	1.4%	1.4%	1.6%	1.4%	1.0%	2.1%	0.6%	0.0%	1.5%	1.5%
		37	*Negativicoccus massiliensis*	0.1%	0.0%	0.0%	0.0%	0.1%	0.0%	0.0%	0.0%	0.1%	0.0%	0.0%	0.0%	0.1%	0.0%	0.0%	0.0%
Proteobacteria	*Enterobacteriaceae*	6	*Klebsiella oxytoca*	0.0%	0.0%	0.0%	0.0%	0.0%	0.0%	0.0%	0.0%	0.0%	0.0%	0.0%	0.0%	0.0%	0.0%	0.0%	0.0%
	Pseudomonadaceae	43	*Pseudomonas aeruginosa*	0.1%	0.1%	0.3%	0.2%	0.0%	0.0%	0.3%	0.1%	0.1%	0.2%	0.3%	0.2%	0.0%	0.0%	0.1%	0.1%
	Sutterellaceae	38	*Sutterella* sp.	0.3%	0.1%	0.0%	0.1%	0.2%	0.1%	0.2%	0.1%	0.2%	0.1%	0.2%	0.1%	0.1%	0.0%	0.1%	0.1%

Table S5: Proportional microbial composition at the family level (%) as determined via 16S-targeted Illumina sequencing in the mucus of the proximal and distal colon compartments of the M-SHIME® before (d14) and after (d16, d26, and d35) treatment with lactose, 2’-FL, 2’-FL/LNnT or HMO6. The intensity of the shading indicates the absolute abundance, normalized for each of the different families within each row.

	Phylum	Family	Lactose				2'-FL				2'-FL/LNnT				HMO6			
			d14	d16	d26	d35	d14	d16	d26	d35	d14	d16	d26	d35	d14	d16	d26	d35
Proximal colon (PC)	Actinobacteria	*Bifidobacteriaceae*	16.3%	14.0%	17.0%	61.3%	15.9%	55.4%	51.2%	10.8%	13.8%	26.1%	41.9%	41.9%	13.2%	21.2%	25.0%	37.9%
		Coriobacteriaceae	0.2%	0.2%	0.2%	0.1%	0.5%	0.5%	0.7%	0.2%	0.1%	0.2%	0.5%	0.1%	0.2%	0.4%	0.2%	0.5%
	Bacteroidetes	*Bacteroidaceae*	1.0%	1.2%	0.4%	2.2%	1.7%	1.0%	0.5%	0.7%	1.8%	1.1%	0.6%	0.5%	1.7%	1.0%	1.4%	1.3%
		Porphyromonadaceae	0.2%	0.3%	0.3%	0.7%	0.4%	0.2%	0.2%	0.6%	0.2%	0.3%	0.2%	0.6%	0.3%	0.5%	0.2%	0.6%
		Rikenellaceae	0.1%	0.1%	1.1%	0.2%	0.3%	0.1%	0.7%	1.1%	0.0%	0.0%	0.6%	0.2%	0.1%	0.1%	0.1%	0.3%
	Firmicutes	*Clostridium* cluster I	1.2%	0.3%	0.0%	0.0%	1.1%	0.5%	0.0%	0.0%	1.0%	0.1%	0.0%	0.0%	0.9%	0.4%	0.0%	0.0%
		Clostridium cluster XI	0.4%	0.1%	0.2%	2.9%	1.3%	0.3%	0.0%	6.9%	0.4%	0.0%	10.3%	0.0%	0.7%	0.0%	5.0%	0.5%
		Lachnospiraceae	16.9%	25.4%	38.8%	13.0%	15.6%	28.0%	15.8%	8.7%	17.1%	10.2%	20.9%	21.0%	17.7%	16.6%	34.0%	22.2%
		Lactobacillaceae	0.0%	0.0%	0.0%	0.0%	0.0%	0.0%	0.0%	0.0%	0.0%	0.0%	0.0%	0.0%	0.0%	0.0%	0.0%	0.0%
		Ruminococcaceae	1.7%	1.3%	0.5%	0.5%	1.6%	1.6%	0.6%	0.5%	1.0%	0.9%	0.7%	0.6%	2.0%	1.3%	1.6%	1.0%
		Veillonellaceae	59.3%	54.1%	37.9%	17.8%	57.9%	10.3%	27.8%	67.9%	61.0%	58.9%	22.1%	32.1%	59.7%	55.3%	30.4%	32.4%
	Proteobacteria	*Enterobacteriaceae*	0.0%	0.0%	0.0%	0.0%	0.0%	0.0%	0.0%	0.0%	0.0%	0.0%	0.0%	0.1%	0.0%	0.0%	0.0%	0.0%
		Pseudomonadaceae	0.0%	0.0%	0.0%	0.0%	0.1%	0.0%	0.0%	0.0%	0.0%	0.0%	0.0%	0.2%	0.0%	0.0%	0.0%	0.0%
		Sutterellaceae	0.3%	0.2%	0.2%	0.1%	0.3%	0.4%	0.2%	0.2%	0.2%	0.1%	0.2%	0.1%	0.2%	0.3%	0.1%	0.1%
Distal colon (DC)	Actinobacteria	*Bifidobacteriaceae*	13.4%	9.8%	10.7%	26.8%	40.1%	20.6%	67.5%	34.3%	7.8%	9.5%	50.1%	22.1%	11.0%	7.9%	51.4%	56.3%
		Coriobacteriaceae	0.1%	0.1%	0.1%	0.1%	1.2%	0.2%	0.6%	0.6%	0.1%	0.1%	0.2%	0.3%	0.0%	0.1%	0.4%	0.2%
	Bacteroidetes	*Bacteroidaceae*	1.0%	1.1%	0.2%	0.7%	0.4%	0.7%	0.3%	0.6%	0.6%	0.7%	0.4%	0.8%	0.4%	0.8%	0.6%	0.7%
		Porphyromonadaceae	0.3%	0.2%	0.2%	0.4%	0.2%	0.2%	0.1%	0.2%	0.1%	0.1%	0.1%	0.2%	0.2%	0.1%	0.4%	0.2%
		Rikenellaceae	0.1%	0.2%	0.5%	0.6%	0.2%	0.1%	0.3%	0.5%	0.0%	0.0%	0.1%	0.2%	0.0%	0.1%	0.1%	0.0%
	Firmicutes	*Clostridium* cluster I	0.4%	0.3%	0.0%	0.1%	0.0%	0.3%	0.0%	0.1%	0.5%	0.3%	0.0%	0.0%	0.4%	0.3%	0.0%	0.1%
		Clostridium cluster XI	24.8%	15.2%	5.1%	0.3%	2.1%	13.9%	0.1%	23.3%	10.6%	13.0%	0.0%	10.4%	7.2%	2.7%	0.0%	3.9%
		Lachnospiraceae	15.1%	12.4%	18.2%	28.5%	5.8%	18.7%	12.7%	17.6%	11.6%	20.0%	23.0%	34.0%	14.1%	12.6%	27.3%	16.5%
		Lactobacillaceae	0.0%	0.0%	0.0%	0.0%	0.0%	0.0%	0.0%	0.0%	0.0%	0.0%	0.0%	0.0%	0.0%	0.0%	0.0%	0.0%
		Ruminococcaceae	1.4%	1.6%	0.4%	0.5%	0.3%	1.3%	0.4%	0.5%	0.8%	0.5%	1.4%	1.5%	0.9%	1.0%	0.9%	0.6%
		Veillonellaceae	40.8%	57.0%	62.9%	39.2%	47.8%	42.2%	16.5%	20.5%	66.1%	53.6%	23.0%	28.8%	63.8%	71.7%	17.1%	20.0%
	Proteobacteria	*Enterobacteriaceae*	0.0%	0.0%	0.0%	0.0%	0.0%	0.0%	0.0%	0.0%	0.0%	0.0%	0.0%	0.0%	0.0%	0.0%	0.0%	0.0%
		Pseudomonadaceae	0.0%	0.0%	0.0%	0.0%	0.2%	0.0%	0.0%	0.0%	0.0%	0.0%	0.0%	0.0%	0.0%	0.0%	0.0%	0.0%
		Sutterellaceae	0.3%	0.2%	0.1%	0.2%	0.1%	0.1%	0.1%	0.1%	0.1%	0.1%	0.1%	0.0%	0.1%	0.1%	0.0%	0.0%

Table S6: Proportional microbial composition at the OTU level (%) as determined via 16S-targeted Illumina sequencing in the mucus of the proximal colon compartment of the M-SHIME® before (d14) and after (d16, d26, and d35) treatment with lactose, 2′-FL, 2′-FL/LNnT or HMO6. The intensity of the shading indicates the absolute abundance, normalized for each of the different OTUs within each row.

Phylum	Family	OTU number	Related species	Mucus - PC															
				Lactose				2'-FL				2'-FL/LNnT				HMO6			
				d14	d16	d26	d35	d14	d16	d26	d35	d14	d16	d26	d35	d14	d16	d26	d35
Actinobacteria	*Bifidobacteriaceae*	2	*Bifidobacterium longum*	6.7%	4.4%	11.2%	2.5%	3.5%	5.3%	0.6%	1.7%	5.5%	4.4%	0.6%	0.2%	4.1%	3.8%	1.5%	0.8%
		3	*Bifidobacterium adolescentis*	6.8%	5.6%	0.7%	54.0%	10.9%	44.9%	44.0%	6.4%	4.6%	10.5%	35.3%	35.3%	3.4%	13.8%	16.1%	29.5%
		9	*Bifidobacterium bifidum*	2.8%	3.9%	5.1%	3.6%	1.5%	4.5%	6.2%	2.5%	3.7%	10.6%	5.8%	6.3%	5.8%	3.6%	7.1%	6.6%
		35	*Bifidobacterium dentium*	0.0%	0.0%	0.0%	0.9%	0.0%	0.1%	0.3%	0.0%	0.0%	0.0%	0.2%	0.0%	0.0%	0.0%	0.0%	0.5%
		44	*Bifidobacterium bifidum*	0.0%	0.0%	0.0%	0.4%	0.0%	0.7%	0.1%	0.2%	0.0%	0.6%	0.1%	0.1%	0.1%	0.0%	0.4%	0.4%
	Coriobacteriaceae	29	*Collinsella aerofaciens*	0.2%	0.2%	0.2%	0.1%	0.5%	0.5%	0.7%	0.2%	0.1%	0.2%	0.5%	0.1%	0.2%	0.4%	0.2%	0.5%
Bacteroidetes	*Bacteroidaceae*	22	*Bacteroides fragilis*	0.1%	0.2%	0.1%	0.6%	0.1%	0.2%	0.1%	0.2%	0.2%	0.2%	0.2%	0.2%	0.2%	0.3%	0.4%	0.4%
		21	*Bacteroides xylanisolvens*	0.5%	0.7%	0.2%	1.1%	0.9%	0.5%	0.2%	0.2%	1.0%	0.6%	0.2%	0.2%	1.0%	0.4%	0.6%	0.6%
		45	*Bacteroides thetaiotaomicron*	0.2%	0.2%	0.0%	0.2%	0.2%	0.1%	0.1%	0.1%	0.4%	0.2%	0.1%	0.1%	0.3%	0.1%	0.1%	0.2%
		50	*Bacteroides caccae*	0.0%	0.0%	0.0%	0.1%	0.1%	0.1%	0.1%	0.0%	0.2%	0.1%	0.1%	0.0%	0.1%	0.1%	0.1%	0.0%
		48	*Bacteroides uniformis*	0.1%	0.1%	0.0%	0.1%	0.2%	0.1%	0.1%	0.1%	0.0%	0.1%	0.1%	0.0%	0.1%	0.1%	0.1%	0.1%
	Porphyromonadaceae	26	*Parabacteroides distasonis*	0.2%	0.3%	0.3%	0.7%	0.4%	0.2%	0.2%	0.6%	0.2%	0.3%	0.2%	0.6%	0.3%	0.5%	0.2%	0.6%
	Rikenellaceae	31	*Allistipes finegoldii*	0.1%	0.1%	1.1%	0.2%	0.3%	0.1%	0.7%	1.1%	0.0%	0.0%	0.6%	0.2%	0.1%	0.1%	0.1%	0.3%
Firmicutes	Clostridium cluster I	46	*Clostridium cadaveris*	1.2%	0.3%	0.0%	0.0%	1.1%	0.5%	0.0%	0.0%	1.0%	0.1%	0.0%	0.0%	0.9%	0.4%	0.0%	0.0%
	Clostridium cluster XI	12	*Sporanaerobacter acetigenes*	0.4%	0.1%	0.2%	2.9%	1.3%	0.3%	0.0%	6.9%	0.4%	0.0%	10.3%	0.0%	0.7%	0.0%	5.0%	0.5%
	Lachnospiraceae	7	*Clostridium clostridioforme*	5.8%	9.3%	22.5%	3.2%	4.4%	6.7%	3.2%	3.9%	4.5%	2.9%	5.1%	4.6%	2.5%	3.7%	4.4%	2.8%
		10	*Roseburia inulinivorans*	2.1%	3.4%	3.9%	2.8%	1.9%	2.3%	1.4%	0.9%	5.9%	1.5%	2.3%	5.0%	6.5%	2.3%	16.7%	10.9%
		8	*Ruminococcus torques/faecis*	0.9%	2.6%	6.1%	3.3%	0.9%	7.3%	7.1%	0.6%	0.8%	1.0%	9.2%	7.0%	1.3%	2.8%	7.2%	3.1%
		42	*Eubacterium rectale*	0.0%	0.0%	0.0%	0.0%	0.0%	0.0%	0.1%	0.0%	0.0%	0.0%	0.0%	0.3%	0.0%	0.5%	0.5%	0.3%
		18	Butyrate-producing bacterium SS3/4	1.5%	2.0%	2.9%	0.3%	1.3%	1.5%	1.7%	0.3%	0.9%	0.6%	0.7%	0.8%	0.8%	2.1%	0.8%	0.9%
		27	*Faecalicatena fissicatena*	2.4%	0.4%	0.1%	0.6%	2.5%	0.7%	0.0%	1.7%	0.6%	0.0%	0.8%	0.0%	1.6%	0.3%	0.3%	0.0%
		19	Butyrate-producing bacterium SR1/5	0.7%	3.6%	0.6%	0.3%	0.9%	3.5%	0.5%	0.3%	0.3%	1.5%	0.2%	0.6%	0.6%	0.9%	0.7%	0.6%
		24	*Clostridium scindens*	1.7%	1.6%	1.1%	0.6%	1.7%	1.6%	0.6%	0.2%	1.8%	0.9%	0.6%	0.7%	1.5%	1.1%	0.6%	0.8%
		28	*Eubacterium ramulus*	0.0%	0.0%	0.1%	0.9%	0.0%	0.0%	0.1%	0.1%	0.0%	0.0%	0.1%	0.6%	0.0%	0.0%	1.0%	1.1%
		23	*Lachnoclostridium sp.*	0.7%	0.9%	0.4%	0.3%	0.8%	0.9%	0.4%	0.1%	1.2%	0.6%	0.6%	0.4%	1.2%	0.8%	0.7%	0.4%
		40	*Blautia wexlerae*	0.1%	0.3%	0.0%	0.0%	0.2%	0.9%	0.0%	0.0%	0.1%	0.6%	0.0%	0.0%	0.6%	0.9%	0.0%	0.0%
		41	*Roseburia hominis*	0.4%	0.2%	0.1%	0.1%	0.2%	0.4%	0.0%	0.1%	0.3%	0.2%	0.1%	0.1%	0.6%	0.1%	0.5%	0.3%
		33	*Blautia producta*	0.2%	0.4%	0.3%	0.5%	0.4%	1.0%	0.1%	0.4%	0.1%	0.1%	0.7%	0.2%	0.3%	0.7%	0.5%	0.2%
		32	*Anaerobutyricum hallii*	0.1%	0.2%	0.1%	0.1%	0.1%	0.3%	0.4%	0.1%	0.1%	0.2%	0.2%	0.5%	0.1%	0.1%	0.1%	0.6%
		39	*Coprococcus comes*	0.2%	0.5%	0.5%	0.1%	0.3%	0.9%	0.1%	0.0%	0.4%	0.2%	0.1%	0.1%	0.3%	0.3%	0.0%	0.2%
	Lactobacillaceae	15	*Lactobacillus paracasei*	0.0%	0.0%	0.0%	0.0%	0.0%	0.0%	0.0%	0.0%	0.0%	0.0%	0.0%	0.0%	0.0%	0.0%	0.0%	0.0%
	Ruminococcaceae	20	*Faecalibacterium prausnitzii*	0.2%	0.3%	0.1%	0.2%	0.1%	0.2%	0.0%	0.2%	0.2%	0.3%	0.0%	0.1%	0.3%	0.2%	0.1%	0.4%
		36	*Clostridium* sp.	0.0%	0.0%	0.0%	0.0%	0.1%	0.1%	0.0%	0.0%	0.0%	0.0%	0.0%	0.3%	0.0%	0.0%	1.0%	0.1%
		34	*Oscilibacter massiliensis*	0.7%	0.4%	0.3%	0.1%	1.0%	0.7%	0.5%	0.2%	0.4%	0.3%	0.5%	0.1%	1.1%	0.6%	0.3%	0.4%
		47	*Ruthenibacterium lactatiformans*	0.4%	0.3%	0.0%	0.1%	0.3%	0.4%	0.0%	0.1%	0.2%	0.2%	0.1%	0.0%	0.4%	0.3%	0.1%	0.1%
		54	*Flavonifractor plautii*	0.3%	0.3%	0.1%	0.0%	0.2%	0.2%	0.1%	0.1%	0.2%	0.1%	0.1%	0.1%	0.2%	0.1%	0.1%	0.1%
	Veillonellaceae	1	*Megamonas funiformis*	56.5%	44.6%	34.2%	16.9%	53.8%	9.1%	25.4%	67.1%	58.7%	55.9%	20.8%	30.7%	57.3%	53.3%	29.3%	30.7%
		13	*Veillonella ratti*	2.2%	8.8%	3.2%	0.7%	3.7%	1.1%	2.2%	0.7%	1.8%	2.8%	1.1%	1.3%	1.9%	1.5%	0.9%	1.5%
		37	*Negativicoccus massiliensis*	0.7%	0.6%	0.5%	0.2%	0.5%	0.1%	0.2%	0.1%	0.5%	0.2%	0.2%	0.1%	0.5%	0.6%	0.2%	0.2%
Proteobacteria	*Enterobacteriaceae*	6	*Klebsiella oxytoca*	0.0%	0.0%	0.0%	0.0%	0.0%	0.0%	0.0%	0.0%	0.0%	0.0%	0.0%	0.1%	0.0%	0.0%	0.0%	0.0%
	Pseudomonadaceae	43	*Pseudomonas aeruginosa*	0.0%	0.0%	0.0%	0.0%	0.1%	0.0%	0.0%	0.0%	0.0%	0.0%	0.0%	0.2%	0.0%	0.0%	0.0%	0.0%
	Sutterellaceae	38	*Sutterella* sp.	0.3%	0.2%	0.2%	0.1%	0.3%	0.4%	0.2%	0.2%	0.2%	0.1%	0.2%	0.1%	0.2%	0.3%	0.1%	0.1%

Table S7: Proportional microbial composition at the OTU level (%) as determined via 16S-targeted Illumina sequencing in the mucus of the distal colon compartments of the M-SHIME® before (d14) and after (d16, d26, and d35) treatment with lactose, 2′-FL, 2′-FL/LNnT or HMO6. The intensity of the shading indicates the absolute abundance, normalized for each of the different OTUs within each row.

				Mucus - DC															
Phylum	Family	OTU number	Related species	Lactose				2'-FL				2'-FL/LNnT				HMO6			
				d14	d16	d26	d35	d14	d16	d26	d35	d14	d16	d26	d35	d14	d16	d26	d35
Actinobacteria	*Bifidobacteriaceae*	2	*Bifidobacterium longum*	7.3%	3.0%	7.2%	3.7%	0.7%	2.5%	0.6%	0.2%	2.9%	1.8%	1.3%	0.2%	2.3%	1.7%	2.6%	0.9%
		3	*Bifidobacterium adolescentis*	3.8%	3.9%	0.4%	17.3%	37.0%	10.8%	58.7%	23.2%	2.2%	1.8%	41.2%	9.5%	1.7%	3.6%	41.9%	45.6%
		9	*Bifidobacterium bifidum*	2.3%	2.7%	3.2%	5.2%	2.3%	6.9%	7.7%	10.5%	2.7%	5.9%	7.4%	12.3%	6.9%	2.6%	5.8%	8.2%
		35	*Bifidobacterium dentium*	0.0%	0.0%	0.0%	0.0%	0.1%	0.0%	0.4%	0.2%	0.0%	0.0%	0.0%	0.0%	0.0%	0.0%	0.7%	1.0%
		44	*Bifidobacterium bifidum*	0.0%	0.1%	0.0%	0.5%	0.0%	0.4%	0.1%	0.2%	0.0%	0.0%	0.0%	0.0%	0.1%	0.0%	0.5%	0.7%
	Coriobacteriaceae	29	*Collinsella aerofaciens*	0.1%	0.1%	0.1%	0.1%	1.2%	0.2%	0.6%	0.6%	0.1%	0.1%	0.2%	0.3%	0.0%	0.1%	0.4%	0.2%
Bacteroidetes	*Bacteroidaceae*	22	*Bacteroides fragilis*	0.1%	0.1%	0.1%	0.3%	0.2%	0.2%	0.1%	0.2%	0.1%	0.1%	0.1%	0.2%	0.1%	0.2%	0.2%	0.4%
		21	*Bacteroides xylanisolvens*	0.5%	0.6%	0.0%	0.3%	0.2%	0.3%	0.1%	0.1%	0.3%	0.3%	0.2%	0.3%	0.2%	0.2%	0.4%	0.2%
		45	*Bacteroides thetaiotaomicron*	0.2%	0.3%	0.0%	0.0%	0.0%	0.1%	0.0%	0.1%	0.1%	0.2%	0.0%	0.1%	0.0%	0.2%	0.0%	0.0%
		50	*Bacteroides caccae*	0.1%	0.0%	0.0%	0.0%	0.0%	0.1%	0.0%	0.0%	0.1%	0.1%	0.0%	0.1%	0.1%	0.1%	0.0%	0.0%
		48	*Bacteroides uniformis*	0.2%	0.1%	0.1%	0.1%	0.0%	0.0%	0.1%	0.1%	0.0%	0.1%	0.1%	0.1%	0.0%	0.1%	0.0%	0.1%
	Porphyromonadaceae	26	*Parabacteroides distasonis*	0.3%	0.2%	0.2%	0.4%	0.2%	0.2%	0.1%	0.2%	0.1%	0.1%	0.1%	0.2%	0.2%	0.1%	0.4%	0.2%
	Rikenellaceae	31	*Allistipes finegoldii*	0.1%	0.2%	0.5%	0.6%	0.2%	0.1%	0.3%	0.5%	0.0%	0.0%	0.1%	0.2%	0.0%	0.1%	0.1%	0.0%
Firmicutes	Clostridium cluster I	46	*Clostridium cadaveris*	0.4%	0.3%	0.0%	0.1%	0.0%	0.3%	0.0%	0.1%	0.5%	0.3%	0.0%	0.0%	0.4%	0.3%	0.0%	0.1%
	Clostridium cluster XI	12	*Sporanaerobacter acetigenes*	24.8%	15.2%	5.1%	0.3%	2.1%	13.9%	0.1%	23.3%	10.6%	13.0%	0.0%	10.4%	7.2%	2.7%	0.0%	3.9%
	Lachnospiraceae	7	*Clostridium clostridioforme*	5.2%	4.0%	12.6%	14.0%	1.4%	1.5%	2.3%	1.7%	1.9%	1.2%	4.7%	2.7%	1.1%	1.6%	2.9%	2.6%
		10	*Roseburia inulinivorans*	2.7%	2.4%	1.0%	4.8%	1.1%	7.5%	1.5%	10.2%	5.4%	14.5%	2.6%	22.5%	7.0%	4.7%	11.3%	8.9%
		8	*Ruminococcus torques/faecis*	0.9%	1.3%	1.7%	3.3%	1.0%	2.2%	5.6%	2.2%	0.4%	1.1%	5.9%	4.4%	1.4%	2.1%	5.4%	1.8%
		42	*Eubacterium rectale*	0.0%	0.0%	0.0%	0.0%	0.0%	0.0%	0.2%	0.1%	0.0%	0.1%	3.4%	1.3%	0.0%	0.0%	1.0%	0.2%
		18	Butyrate-producing bacterium SS3/4	0.4%	0.4%	0.5%	1.9%	0.3%	0.3%	1.3%	0.8%	0.3%	0.2%	3.1%	0.8%	0.2%	0.3%	2.1%	0.2%
		27	*Faecalicatena fissicatena*	2.5%	1.0%	1.3%	0.0%	0.3%	0.5%	0.1%	0.8%	1.2%	0.1%	0.0%	0.0%	0.9%	0.1%	0.0%	0.5%
		19	Butyrate-producing bacterium SR1/5	0.5%	1.2%	0.2%	1.2%	0.3%	1.5%	0.4%	0.4%	0.5%	0.5%	0.6%	0.4%	0.6%	0.9%	1.6%	0.3%
		24	*Clostridium scindens*	1.0%	0.6%	0.3%	0.8%	0.1%	0.8%	0.4%	0.4%	0.8%	0.7%	0.3%	0.3%	0.7%	1.0%	0.2%	0.4%
		28	*Eubacterium ramulus*	0.0%	0.0%	0.0%	0.8%	0.3%	0.0%	0.0%	0.4%	0.0%	0.0%	1.2%	0.5%	0.0%	0.0%	1.0%	0.8%
		23	*Lachnoclostridium sp.*	0.5%	0.5%	0.2%	0.5%	0.5%	0.6%	0.5%	0.3%	0.4%	0.4%	0.6%	0.3%	0.6%	0.4%	0.4%	0.1%
		40	*Blautia wexlerae*	0.2%	0.3%	0.0%	0.0%	0.0%	0.6%	0.0%	0.0%	0.1%	0.2%	0.0%	0.0%	0.5%	0.6%	0.0%	0.0%
		41	*Roseburia hominis*	0.3%	0.1%	0.0%	0.3%	0.1%	1.5%	0.1%	0.2%	0.2%	0.8%	0.1%	0.4%	0.6%	0.2%	0.5%	0.3%
		33	*Blautia producta*	0.8%	0.6%	0.3%	0.2%	0.1%	1.4%	0.1%	0.1%	0.2%	0.2%	0.1%	0.2%	0.3%	0.5%	0.1%	0.2%
		32	*Anaerobutyricum hallii*	0.0%	0.1%	0.0%	0.4%	0.3%	0.1%	0.3%	0.1%	0.0%	0.0%	0.2%	0.2%	0.0%	0.0%	0.7%	0.1%
		39	*Coprococcus comes*	0.1%	0.1%	0.0%	0.3%	0.0%	0.1%	0.0%	0.0%	0.2%	0.1%	0.0%	0.0%	0.2%	0.2%	0.1%	0.0%
	Lactobacillaceae	15	*Lactobacillus paracasei*	0.0%	0.0%	0.0%	0.0%	0.0%	0.0%	0.0%	0.0%	0.0%	0.0%	0.0%	0.0%	0.0%	0.0%	0.0%	0.0%
	Ruminococcaceae	20	*Faecalibacterium prausnitzii*	0.1%	0.3%	0.0%	0.2%	0.2%	0.2%	0.1%	0.0%	0.1%	0.1%	0.4%	0.2%	0.2%	0.2%	0.7%	0.3%
		36	*Clostridium* sp.	0.1%	0.1%	0.0%	0.0%	0.0%	0.1%	0.0%	0.0%	0.0%	0.1%	0.8%	0.8%	0.0%	0.0%	0.0%	0.0%
		34	*Oscilibacter massiliensis*	0.6%	0.6%	0.2%	0.2%	0.0%	0.5%	0.2%	0.3%	0.3%	0.2%	0.1%	0.2%	0.4%	0.2%	0.1%	0.1%
		47	*Ruthenibacterium lactatiformans*	0.4%	0.4%	0.1%	0.0%	0.0%	0.5%	0.0%	0.2%	0.2%	0.2%	0.0%	0.2%	0.2%	0.5%	0.0%	0.1%
		54	*Flavonifractor plautii*	0.2%	0.2%	0.0%	0.1%	0.0%	0.1%	0.0%	0.1%	0.2%	0.1%	0.0%	0.1%	0.1%	0.1%	0.0%	0.0%
	Veillonellaceae	1	*Megamonas funiformis*	39.2%	55.4%	61.8%	36.4%	46.5%	41.1%	15.1%	19.9%	65.2%	52.5%	21.4%	28.0%	63.2%	71.6%	16.0%	19.3%
		13	*Veillonella ratti*	1.3%	1.4%	0.8%	2.7%	1.3%	1.0%	1.2%	0.5%	0.7%	0.9%	1.6%	0.6%	0.4%	0.0%	1.0%	0.7%
		37	*Negativicoccus massiliensis*	0.3%	0.2%	0.2%	0.1%	0.0%	0.2%	0.2%	0.1%	0.2%	0.2%	0.1%	0.1%	0.1%	0.2%	0.1%	0.0%
Proteobacteria	*Enterobacteriaceae*	6	*Klebsiella oxytoca*	0.0%	0.0%	0.0%	0.0%	0.0%	0.0%	0.0%	0.0%	0.0%	0.0%	0.0%	0.0%	0.0%	0.0%	0.0%	0.0%
	Pseudomonadaceae	43	*Pseudomonas aeruginosa*	0.0%	0.0%	0.0%	0.0%	0.2%	0.0%	0.0%	0.0%	0.0%	0.0%	0.0%	0.0%	0.0%	0.0%	0.0%	0.0%
	Sutterellaceae	38	*Sutterella* sp.	0.3%	0.2%	0.1%	0.2%	0.1%	0.1%	0.1%	0.1%	0.1%	0.1%	0.1%	0.0%	0.1%	0.1%	0.0%	0.0%
